# The influences of microbial colonisation and germ-free status on the chicken TCRβ repertoire

**DOI:** 10.3389/fimmu.2022.1052297

**Published:** 2023-01-04

**Authors:** Stefan Dascalu, Stephen G. Preston, Robert J. Dixon, Patrik G. Flammer, Steven Fiddaman, Amy Boyd, Joshua E. Sealy, Jean-Remy Sadeyen, Bernd Kaspers, Philippe Velge, Munir Iqbal, Michael B. Bonsall, Adrian L. Smith

**Affiliations:** ^1^ Department of Biology, University of Oxford, Oxford, United Kingdom; ^2^ Avian Influenza Research Group, The Pirbright Institute, Pirbright, United Kingdom; ^3^ UCL School of Pharmacy, University College London, London, United Kingdom; ^4^ Veterinary Faculty, Ludwig Maximillians University of Munich, Planegg, Germany; ^5^ Institut National de la Recherche Agronomique (INRAE), Université François Rabelais de Tours, Unités Mixtes de Recherche, Infectiologie et Santé Publique (ISP), Nouzilly, France

**Keywords:** T cell receptor (TCR), repertoire, chicken, microbiome, germ free

## Abstract

Microbial colonisation is paramount to the normal development of the immune system, particularly at mucosal sites. However, the relationships between the microbiome and the adaptive immune repertoire have mostly been explored in rodents and humans. Here, we report a high-throughput sequencing analysis of the chicken TCRβ repertoire and the influences of microbial colonisation on tissue-resident TCRβ+ cells. The results reveal that the microbiome is an important driver of TCRβ diversity in both intestinal tissues and the bursa of Fabricius, but not in the spleen. Of note, public TCRβ sequences (shared across individuals) make a substantial contribution to the repertoire. Additionally, different tissues exhibit biases in terms of their V family and J gene usage, and these effects were influenced by the gut-associated microbiome. TCRβ clonal expansions were identified in both colonised and germ-free birds, but differences between the groups were indicative of an influence of the microbiota. Together, these findings provide an insight into the avian adaptive immune system and the influence of the microbiota on the TCRβ repertoire.

## Introduction

The interactions between the microbiome and the host are important for many processes such as nutrition, mucosal physiology as well as protection against pathogens *via* competitive exclusion and the stimulation of the immune system (1–4). Among the different compartments of the host microbiome, the gastrointestinal tract deserves special attention, as it harbours the largest microbial abundance and diversity (4, 5). To date, most studies exploring the influence of the gut microbiota on the immune system have focused on rodents and humans (6, 7). It is, therefore, important to consider the impact of the microbiota on immune processes in other species to evaluate if findings from these systems apply more generally.

The domestic chicken (*Gallus gallus domesticus*) represents both a model organism for biological research and an economically important source of protein at a global scale (2, 4, 8). As in mammals, the chicken gut represents a major route for exogenous antigen uptake, and thus is extremely relevant to the normal development of both the innate and adaptive components of immunity (2–4) [for a general overview of avian gut immunology see (9)]. Regarding the latter, the relations between the microbiome and the enormous diversity of unique lymphocyte receptors (i.e. the adaptive immune repertoire) raise important questions, as the chicken gut microbiota has previously been shown to be an important driver of both T and B cell responses (2, 4). From a practical perspective a better understanding of chicken microbiota–T cell interactions is relevant both to gut health and disease resistance which impact on the welfare and efficiency of poultry production.

The processes involved in the diversification of the T cell receptor (TCR) repertoire in birds are similar to those reported in mammals involving RAG-dependent rearrangement with junctional modification. The TCR loci are arranged similarly in birds and mammals although the numbers of TCRVβ segments are fewer in the chicken compared with mammals [reviewed in (10)]. Moreover, the chicken TCRVβ gene segments fall into just three families (called Vβ1, Vβ2 and Vβ3) which is many fewer than seen in any mammal (10). By contrast, the TCRγ locus is expanded compared with most mammals; as such, chickens have a greater number of TCRVγ gene segments although not all Vγ gene segments in the genome are utilised with equal frequency (11). Hence the complexity of the locus does not relate to the available repertoire and to understand the function of chicken T cells, the diversity of TCR needs to be considered. In addition to the limited V gene availability in the TCRβ locus, other features of the avian immune system that may affect the TCRβ repertoire include the relative simplicity of the chicken MHC and the expression of single dominant MHC class I and II genes (12, 13). Therefore, although many similarities exist between the avian and mammalian immune systems, the differences raise interesting questions, especially when considering the diversity of the adaptive immune repertoire and its role in health and disease.

Previous studies of chickens have investigated the influence of germ-free conditions on the immune repertoire using methods such CDR3 length profiles alongside molecular cloning and subsequent Sanger sequencing of products to evaluate TCR identity (14). However, the use of high-throughput sequencing (HTS), offers a unique opportunity for comparisons to be made at the level of individual clones (15–19). Therefore, such HTS approaches can shed light on different mechanisms of immune development, regulation, defence against pathogens, and other factors which underpin these phenomena.

In this study, we applied and optimised an HTS-based methodology using 5’RACE PCRs to assess the effects of the microbiome on the TCRβ receptor diversity in chickens, and the extent to which these influences become apparent in different tissues. We focused on the β chain of the T cell receptor (TCRβ) as it exhibits higher combinatorial potential due to the D gene segment usage and its unique expression on cells *via* allelic exclusion (15, 20–22).

## Materials and methods

### Animal tissue samples and experimental design

Tissue samples (spleen, bursa of Fabricius, jejunum, caecum and colon) were derived from an *in vivo* study carried out at the infectiology platform PFIE (INRA, Val de Loire) in accordance with the national and international regulations specific to the research facility as part of the Development of Immune Function and Avian Gut Health (DIFAGH) consortium. One group of PA12 white leghorn chickens (n=5) were reared under germ-free conditions, whilst another group (n=5) were reared under conventional specific pathogen-free (SPF) conditions. For the germ-free birds, the eggs were collected immediately after laying and surface sterilized by an immersion in 1.5% Divosan Plus VT53 (Johnson Diversey, France) for 5 minutes at room temperature. Subsequently, these eggs were transferred into HEPA-filtered incubator. After 18 days at 37°C, the surface of the eggs was sterilized in 1.25% Divosan for 4 min at 37°C. After hatching, the temperature of the isolator was maintained at 37.5°C for 7 days, then reduced by 1°C per day until reaching a stable temperature of 25°C. Chickens were offered X ray-irradiated starter diet (Special Diets Services; Dietex, Argenteuil, France) and sterilized water ad libitum. The sterility of chickens was confirmed weekly by incubating fresh faecal droppings in 10 mL of sterile brain–heart infusion broth under both aerobic and anaerobic conditions, which allows for bacterial, yeast, and fungal growth. The absence of non-culturable bacteria in faecal and caecal samples of germ-free chickens was confirmed by quantitative PCR of a conserved region of the bacterial ribosomal 16S gene. Individuals from both groups were culled at day 55 post-hatch, when tissue samples were harvested and preserved in RNAlater (Thermo Fisher Scientific) according to manufacturer’s instructions. Samples were stored at –80^0^C prior to processing.

### RNA extraction

Tissue samples were weighed and 15 mg of each sample was placed in 600 µl of RLT lysis buffer (Qiagen) together with 100 µl of 0.2 mm silica beads (Thistle Scientific), and subjected to five cycles of 1-minute homogenisation in a Mini-Beadbeater-24 (BioSpec) and 30 seconds of cooling on ice. RNA was extracted using the RNeasy Mini kit (Qiagen) following the manufacturer’s protocol. For all samples, on-column genomic DNA digestion was performed, using the RNase-Free DNase Set (Qiagen) in accordance with the manufacturer’s instructions. The resulting RNA was eluted in 40 µl of nuclease-free water. The quality and integrity of several samples was tested using an RNA ScreenTape (Agilent Technologies) on the 4200 TapeStation (Agilent Technologies). The RNA samples were stored at –80°C until further processing.

### cDNA generation and 5’RACE PCRs

5’RACE-ready cDNA was generated using the SMARTer kit (Takara), following the manufacturer’s instructions. Subsequently, for each cDNA sample, specific 7-bp barcoded forward primers (5’-NNNNNNNGAAAAGATGACCACATCTGGTTC-3’) for the chicken TCRβ were used for the 5’RACE PCRs. Universal SMARTer kit reverse primers were used for all samples, specific to the common 5’ adapter that was added during 5’RACE cDNA synthesis. Briefly, for each 25 µl reaction, 5 µl of Phusion 5X Buffer (New England Biolabs), 0.5 µl of 10 mM dNTP, 0.5 µl of 10 µM UPA-short primer, 0.5 µl of 2 µM UPA-long and 0.25 µl Phusion Hot Start Flex DNA Polymerase (New England Biolabs) were added to 15.25 µl nuclease-free water, for a total of 22 µl volume. To this, 0.5 µl of the 10µM gene-specific 7 bp-barcoded primer and 2.5 µl of cDNA were added. The individual 25 µl volume 5’RACE PCR reactions were then carried out in 96-well plates using the thermocycler program recommended by the 5’RACE kit (Takara), with 35 cycles of gene-specific amplification with an annealing temperature of 60°C. After PCR amplification, barcoded samples were pooled and subjected to electrophoresis on a 1.4% agarose in Tris/Borate/EDTA (TBE) buffer gel containing 1:10,000 SYBR green (Sigma-Aldrich) at 120 V for 35 minutes. The bands of the expected lengths were gel extracted and purified using the QIAquick Gel Extraction Kit (Qiagen).

### DNA Library preparation and sequencing

DNA libraries of the pooled barcoded PCR samples were generated using the NEBNext Ultra II DNA Library Prep Kit for Illumina (New England Biolabs), following the manufacturer’s instructions. Briefly, this involved the ligation of library adapters, excision, PCR enrichment, and clean up using AMPure XP beads. The quantity and quality of the DNA libraries were analysed using the NEBNext Library Quant Kit for Illumina (New England Biolabs) and a D1000 DNA tape (Agilent Technologies) on the 4200 TapeStation (Agilent Technologies). The libraries were sequenced using an Illumina MiSeq platform at the Department of Biology, University of Oxford.

### Sequence data processing and analysis

The raw sequence data was processed by using an in-house python package (available on GitHub: https://github.com/sgp79/reptools) (23). Briefly, the software assigns the V and J gene IDs to the sequences by BLAST (24) comparing them to a database of known reference sequences. Subsequently, the algorithm extracts the CDR3 after a Smith-Waterman alignment (25), thus allowing for higher precision at the junctions (26). The output was then analysed using *R*, as described below (27).

### Linear mixed-effects models

Linear mixed-effects models were constructed to assess the tissue-specific contribution of TCRβ clones from the microbial treatment groups. In order to account for individual-specific variability, the effect of each bird was incorporated as a random intercept. The parameters, tissue and status (i.e. treatment group), were incorporated as explanatory variables in all the models.

Due to the proportional nature of the observations, several data transformations were compared in order to select the most appropriate model. Following exploratory analyses which examined the normality of residuals and homoscedasticity, logit transformations were used when the data were represented by proportions (28). Square root transformations were applied for the Hill numbers used to analyse the repertoire diversity. The models were implemented using the *lme4* package in *R* using Satterthwaite’s approximation, with p values and 95% bootstrap confidence intervals for the model estimates being computed using the *lmerTest* package (29, 30).

#### Repertoire diversity

The suggested patterns of expansion with respect to the tissue type and microbial colonisation status were further investigated by analysing the diversity present within the samples. For this, the effective number of clones (*D)* was calculated using the weighted abundance of unique reads in each sample (31, 32). Corresponding values for species richness (D_0_ – defined here as “clonal richness”), the exponential of the Shannon entropy (D_1_ – defined here as “typical clones”) and the inverse Simpson concentration (D_2_ – defined here as “dominant clones”) diversity measures were computed, thus incorporating the effect of clonal expansions at different levels. This was achieved using the iNEXT package (33) in R, and interpolation or extrapolation was carried out in order to standardise between samples at a value of 10,000 total reads (33). Individual measurements were then incorporated into a linear mixed effects model, with the *response* representing the estimated effective number of species at the particular diversity measurements (D_0_, D_1_, or D_2_) for the samples, as a function of microbial colonisation status and tissue type.

#### Public and private clonal compartments

Based on their CDR3 nucleotide sequence, V family, and J gene usage, clones were divided into the private or public compartments if they were found shared between individuals. The percent contribution to the private or public clonal compartment was estimated as a function of microbial treatment group, tissue type, and clonal compartment (private or public). At first, all clones which were found in more than one individual were regarded as public, irrespective of the number of individuals which shared them. Subsequently, a new model was constructed where public clones were divided into multiple categories based on their presence across the 10 birds: rare publics (between 2 and 5 birds), common publics (between 5 and 9), and ubiquitous clones (shared between all individuals).

#### V family and J gene usage

The contribution of the V gene families and the J genes to the TCRβ repertoire was assessed with regards to the microbial colonisation status and tissue identity. Since there is a high sequence similarity (>90%) within each V family (10), a significant proportion (close to 10%) of the identified reads could not be identified and assigned correctly with a specific V gene within the respective families. In order to overcome this issue, only the V family identity was used for the analysis. There were no assignment issues with regard to the J genes, as out of the total sequences only 12 reads were ambiguously assigned, and the corresponding clones were disregarded from the analysis. Subsequently, an extension of this model was used to calculate the mean estimates for V family and J gene usage in the private and public clonal compartments. For this, two versions of the model were incorporated. First, the private and the total public compartment were used. Subsequently, the public compartment was divided based on different degrees of clonal sharing between birds. Both models incorporated *clonal compartment* as an additional explanatory variable for the publicness of clones.

## Results

### Recovered sequences and productively rearranged TCRβ chains

**Figure 1:**
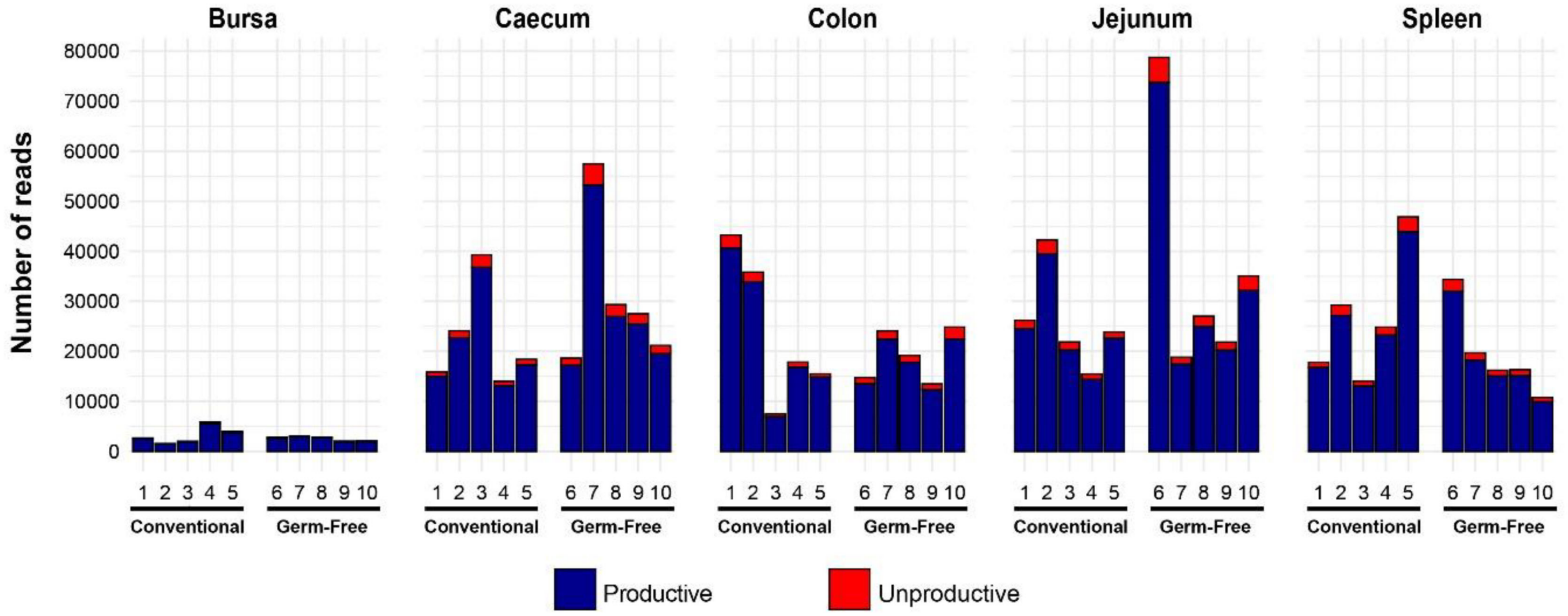
Total chicken TCRβ reads identified in different tissues of chickens reared under germ-free or conventional (SPF) conditions. Bird numbers and their corresponding microbial colonisation status are displayed on the x axis. Productive and unproductive reads are shown in blue and red, respectively.

Following library generation and sequencing, a total of 1,052,190 TCRβ reads were obtained, of which 980,606 (~93.2%) were productively rearranged (in frame with no premature stop codons) (**Figure 1**). Importantly, within tissue type, there were no apparent differences in the total reads acquired or the proportions of productive reads between the germ-free and conventional birds. However, high levels of heterogeneity were observed between different tissues in terms of total sequences. Although the bursa is considered an organ associated with B cell diversification, this site does include T cells and was therefore included in the analyses (34, 35). Of note, substantially fewer TCRβ sequences were recovered from the bursa than from the other tissues. By contrast, the other tissue samples provided a much higher number of productive reads per sample (average of 24,000). For the subsequent parts of the analysis, only the productively rearranged TCRβ sequences were examined.

### Tissue-specific clonal homeostasis

In order to examine the T cells in each tissue, clonal homeostasis plots were generated using the proportion of individual clones within each sample. When the proportions between two or more clones in the sample were equal (i.e. a tied rank), the highest rank among them was assigned, and the next rank position(s) were skipped. This method was chosen to emphasise the differences in abundance between the clones, whilst still capturing the diversity of the clonotype composition. As such, patterns were revealed based both on tissue type and the microbial colonisation status.

**Figure 2:**
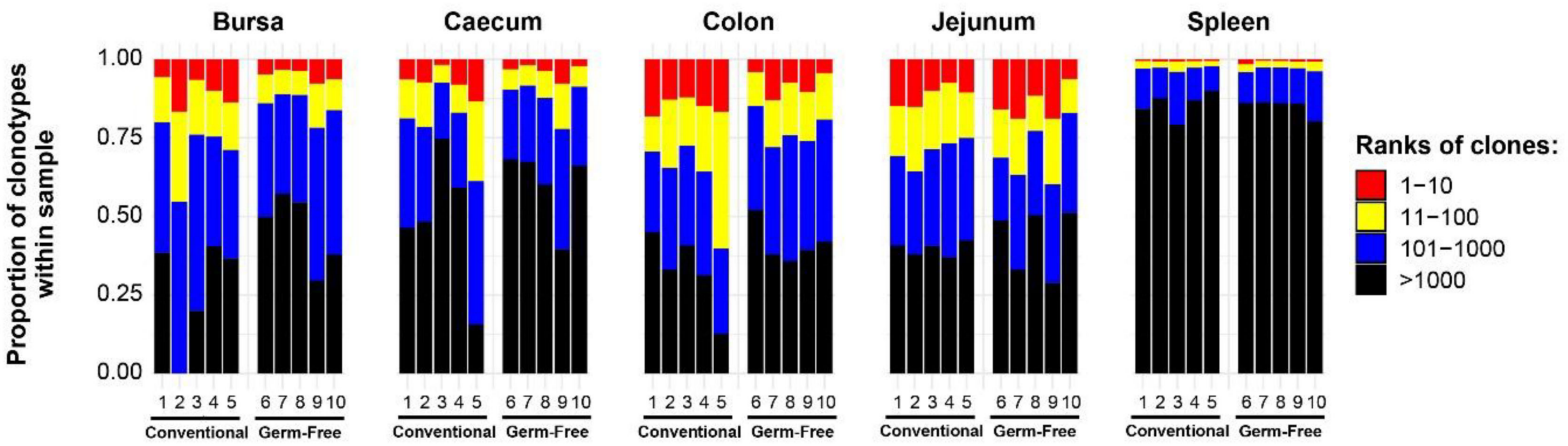
TCRβ clonal homeostasis plots of individual tissue samples. Bird numbers and their corresponding microbial colonisation status are displayed on the x axis. Clones were ranked based on their abundance into four categories: first 10 most abundant (red), from 11-100 (yellow), 101-1000 (blue), and above 1000 (black) in terms of total abundance within each sample. The proportions of clonotypes are displayed on the y axis.

Broadly, the intestinal tissues and the bursa exhibit greater proportions of clonal expansions than the spleen (**Figure 2**). This is indicated by the proportion of the total being represented by the most abundant ranked clones. The pattern in the spleen with lower ranked clones making up >75% of total reads may reflect a large proportion of unexpanded (naïve) T cells (noting that chickens have no lymph nodes). Since read numbers across tissues (apart from the bursa) were broadly comparable, the patterns of expansion were not simply a product of systematically different sequencing depth or coverage.

When comparing the tissue-specific repertoires between the treatment groups, the spleen and jejunum exhibited no marked differences between germ-free and conventional groups. Conversely, the colon, caecum, and bursa seem to have more clonal expansions (a greater proportion of the total made up by the highest ranked clones) in the conventional chickens than in the germ-free group.

### Tissue-specific repertoire diversity

**Figure 3:**
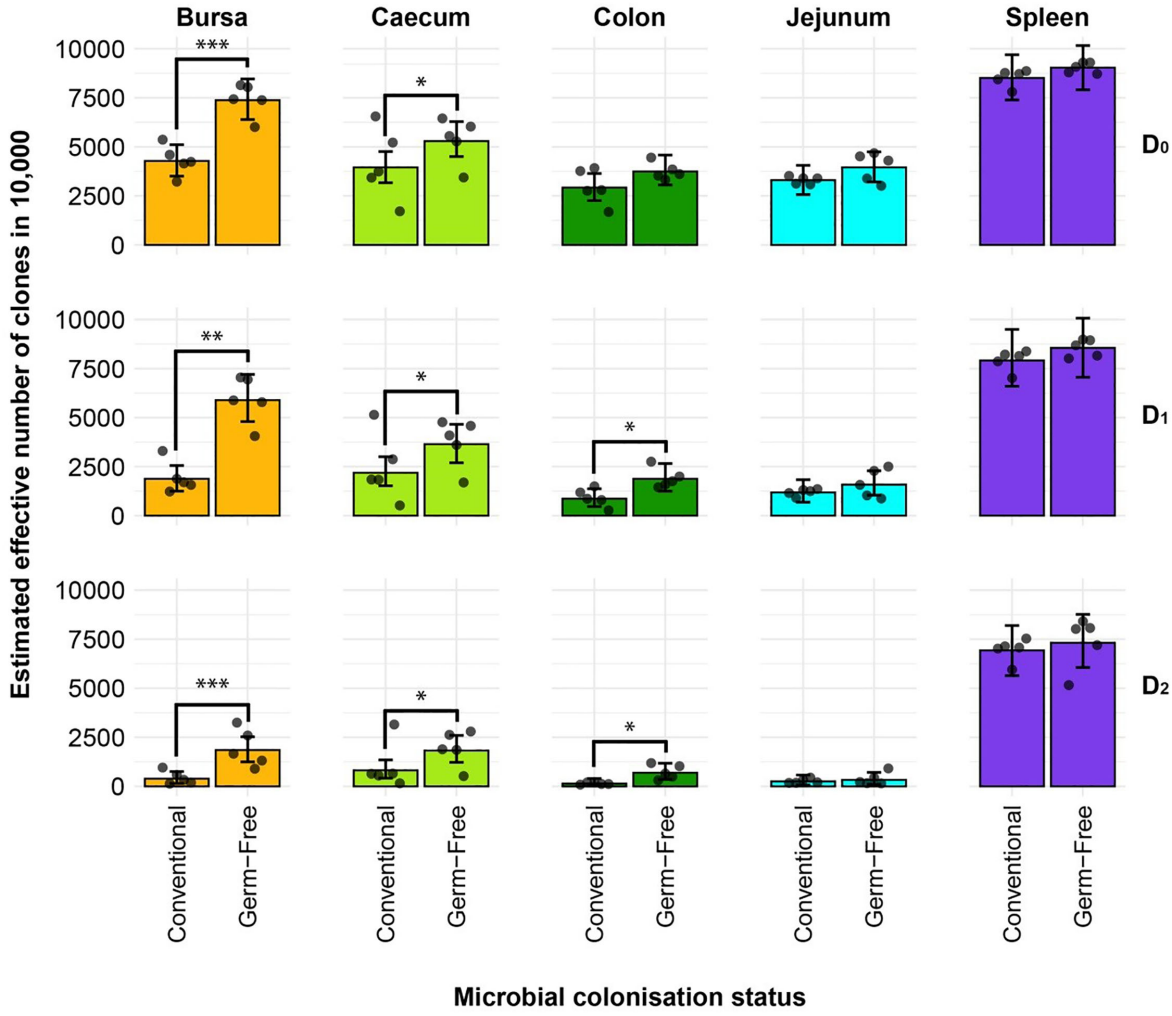
Effective diversity within conventional and germ-free samples. Different rows show the effective number of clones corresponding to clonal richness (D_0_), the typical clones (D_1_), and dominant clones (D_2_). Tissues are colour coded for the bursa (orange), caecum (green), colon (dark green), jejunum (light blue), and spleen (purple). Dots represent individual bird observations of the effective number of species calculated in each tissue for the corresponding Hill number values. Error bars show the 95% bootstrap confidence intervals for the point estimates generated from 1000 simulations of the model. Statistically significant differences between the model estimates are depicted above the plots based on their corresponding p-values: *p < 0.05; **p < 0.01, ***p < 0.001.

The diversity analysis offers valuable insights into the TCRβ clonal diversity of each tissue type between the two treatment groups (**Figure 3**). In terms of clonal richness (D_0_), the repertoire of germ-free chickens is significantly more diverse in the bursa and caecum, whilst there is no difference compared to the conventional birds in the other tissues. However, when “typical clones” are considered (exponential of the Shannon entropy: D_1_), the difference between the colon samples also becomes significant, with the germ-free being more diverse than the conventional birds. Although the effective number of species is lower, these patterns are also maintained for the “dominant clones” (inverse Simpson concentration: D_2_).

The large intestines (caecum and colon) and bursa of conventional birds were less diverse in terms of both the number of clones present (caecum and bursa) and the typical and dominant clones in the tissues (colon, caecum, and bursa), as suggested by the D_1_ and D_2_ measurements, respectively. By contrast, the spleen and jejunum samples exhibit no such pronounced differences, suggesting that microbial colonisation does not influence the repertoire diversity in those tissues. These results strongly indicate that microbial colonisation is a driver of clonal expansions within the resident TCRβ repertoire, particularly in the intestinal tissues where the highest microbial loads are present. Comparable patterns of diversity with the same significant differences (between tissues and/or treatment groups) were observed when considering the repertoires at the amino acid level (**Supplementary Figure 1**).

### Public and private clonal compartments

**Figure 4:**
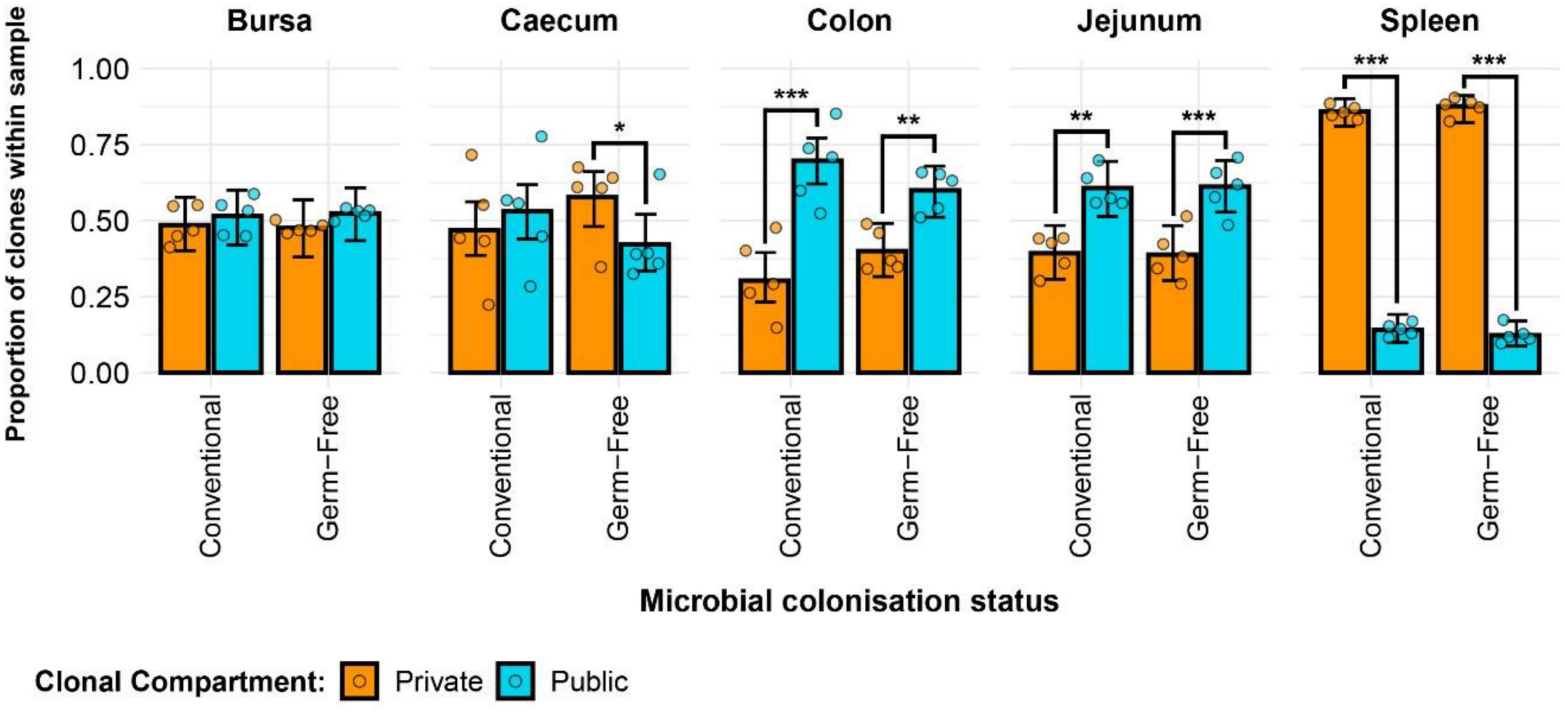
TCRβ clone CDR3 nucleotide public and private compartments. Private (individual-restricted) clones are shown in orange. Public clones (shared between more than two individuals) and are shown in light blue. Dots represent individual bird observations of public and private clonal compartments. Error bars represent 95% bootstrap confidence intervals for the point estimates generated from 1000 simulations of the model. Statistically significant differences between the model estimates are depicted above the plots based on their corresponding p-values: *p < 0.05; **p < 0.01, ***p < 0.001.

Clonal sharing across individuals is a key aspect of the T cell repertoire that could be used to predict the possibility of shared responses based upon repertoire. The public clonal compartment constitutes a dominant component of the repertoire in all tissues, with the exception of the spleen where it was found at a lower but still pronounced level (**Figure 4**). In the intestinal segments, the proportion of public clones generally exceeded 45% of the total identified TCRβ clones, sometimes reaching almost 75% in the jejunum and colon. Interestingly, there were no obvious differences between the germ-free and conventional chickens (**Supplementary Figure 2**). As expected, the private clonal compartment in the spleen is at significantly higher levels than the public clones, amounting close to 90% of sequences. By contrast, in the caecum and bursa, the two compartments were not significantly different to one another, with the exception of the germ-free caecal samples which had a higher proportion of private clones. In the colon and jejunum, however, the public clones were significantly more abundant, encompassing more than half of the total clonal compartment.

At the amino acid level (**Supplementary Figure 3**), the patterns were comparable with a slightly higher proportion of public clones being identified. This resulted in significantly higher proportions of public rather than private clones in the bursal compartments of both treatment groups. Additionally, in the caecum, the previously identified significant difference between the public and private clones in the germ-free was no longer observed due to the higher proportion of public clones at the amino acid level. Furthermore, the conventional birds exhibited significantly higher proportions of public rather than private clones in the caecum when the CDR3 amino acid rather than nucleotide sequences were considered. No differences were observed between the groups when individual tissues were considered (**Supplementary Figure 4**).

**Figure 5:**
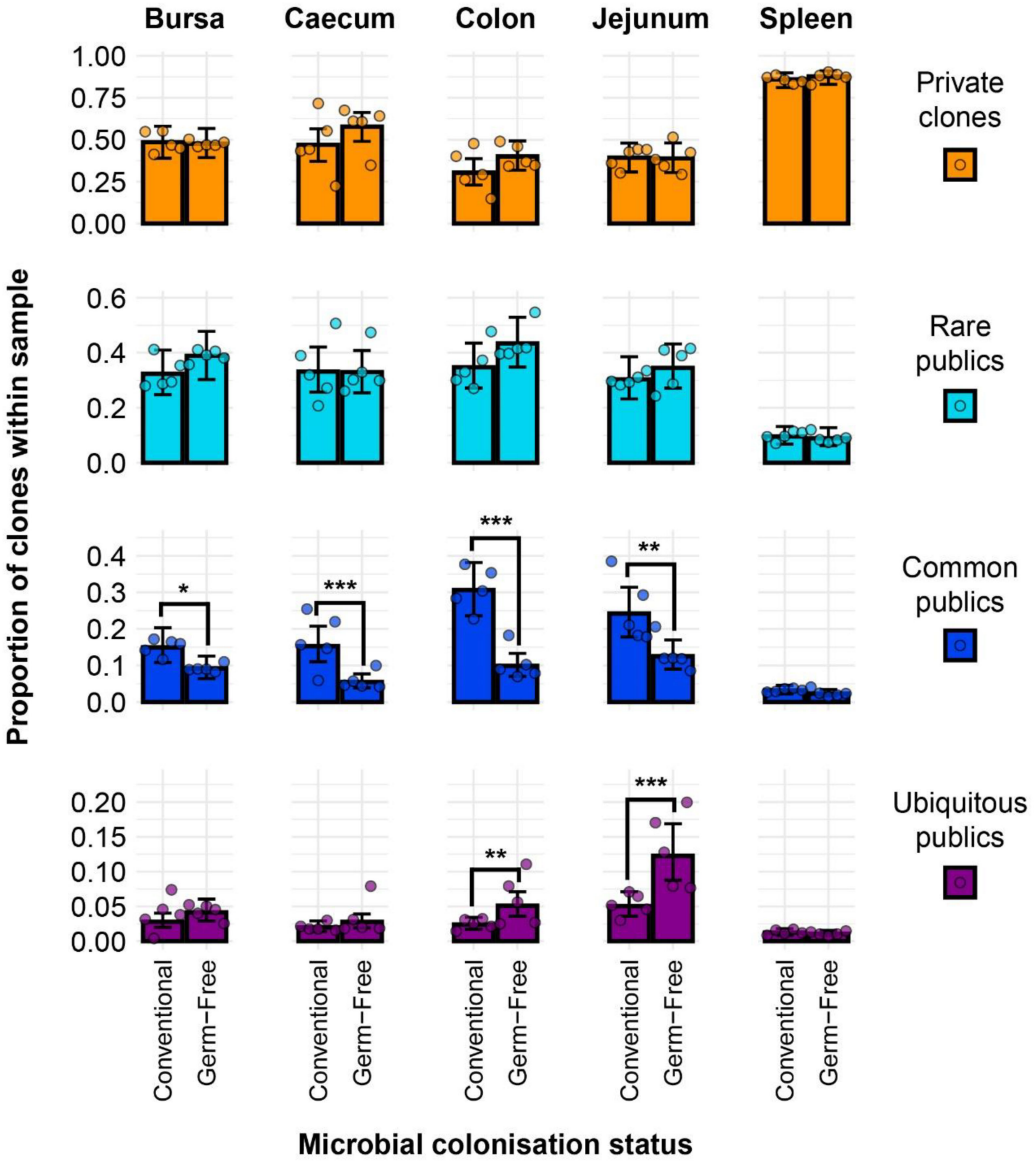
TCRβ clone CDR3 nucleotide private and public compartments based on different levels of clonal sharing between birds. Private (individual-restricted) clones are shown in orange. Rare publics (shared between ≥2 individuals and up to 5) and are shown in light blue. Common publics (shared between ≥5 and up to 9 birds) are shown in dark blue. Ubiquitous publics (found in all birds which were incorporated in the analysis) are shown in purple. Dots represent individual bird observations of private and distinct public clonal compartments. Error bars represent 95% bootstrap confidence intervals for the point estimates generated from 1000 simulations of the model. Statistically significant differences between the model estimates are depicted above the plots based on their corresponding p-values: *p < 0.05; **p < 0.01, ***p < 0.001.

As the publicness of clones, in the context of the TCR repertoire, is a relative term, it is important to discriminate between different degrees of clonal sharing (i.e. how many individuals share a particular clone of TCRβ). When incorporating the different categories of public clones into the model, differences become apparent between the two microbial treatment groups when specific public classes are considered (**Figure 5**). As expected, the clones with higher degrees of publicness occupy less of the total clonal compartments within tissues. Public clones which are found in fewer than five birds seem to dominate the total clonal composition with no significant differences between the two microbial groups. However, when considering the ‘common publics’ (i.e. found in more than five birds but not in all birds of the analysis), all tissues aside from the spleen showed a significantly higher proportion in the conventional microbiota birds. Interestingly, ubiquitous clones which are present in all birds can be identified, albeit at a low overall proportion of the total population. The highest proportions of ubiquitous clones were recorded in the jejunum and caecum, where the germ-free samples have significantly higher levels than their conventional counterparts. Similar patterns were observed when considering the level of CDR3 amino acids (**Supplementary Figure 5**), with the notable exception of the caecum, where the germ-free birds exhibited significantly higher proportions of private clones than the conventional birds.

**Figure 6:**
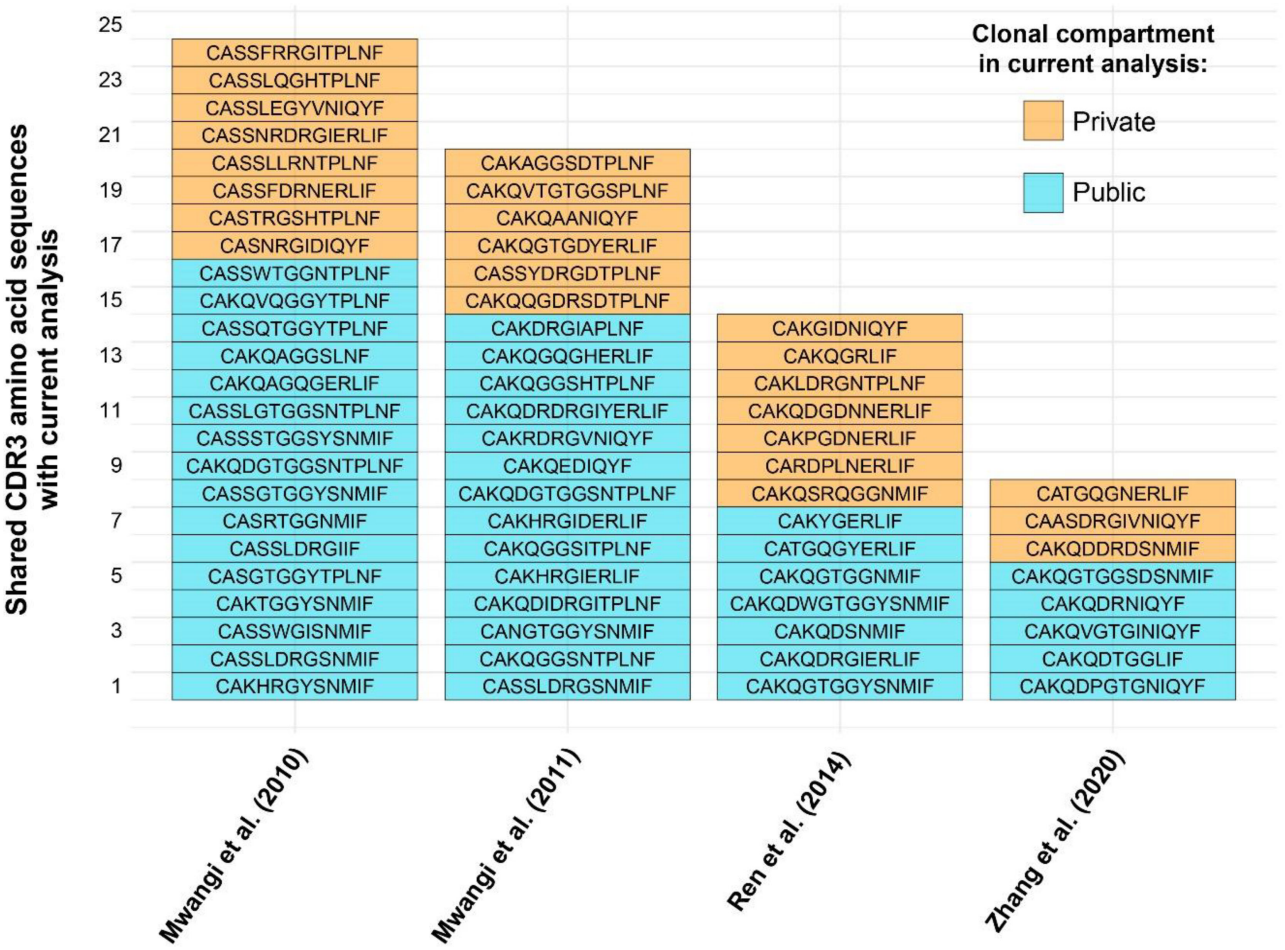
TCRβ shared CDR3 amino acid sequences with other published works. Sequence identity based on clonal compartments within current analysis is colour coded for the private (individual-restricted) and public (shared between two or more birds) repertoires in orange and light blue, respectively.

The high proportion of public clones in the samples warranted further investigation. Cross-contamination due to experimental error was excluded as a possibility given that the samples from the two treatment groups were processed and sequenced separately, and precautions were taken to prevent cross-contamination between individual tissue samples. Moreover, TCRβ CDR3 sequences reported in other published works (also using different lines of chicken) were compared at the amino acid and, where available, nucleotide levels with the sequencing results of the current analysis. As such, several amino acid sequences from previously published works of lower sequencing depth were identified in the current dataset (**Figure 6**). As such, 24/97 (~25%) CDR3 amino acid sequences from Mwangi et al., 2010 (14), 20/96 (~21%) from Mwangi et al., 2011 (36), 14/275 (~5%) from Ren et al., 2014 (37), and 8/119 (~7%) from (38, 39) were found to be shared with the current dataset. Interestingly, not all cross-study shared CDR3 sequences were from the public clonal compartment of the current microbial colonisation experiment. This suggests that the ‘true public compartment’ may be larger than the present analysis was able to identify.

As single amino acids may be coded for by distinct codons, when considering the nucleotide sequences, fewer CDR3s are present in other reported datasets (**Table 1**), the majority of which are found in Mwangi et al., 2010. Out of the shared sequences, none were identified which are shared between all birds of the current analysis. However, whilst most shared CDR3s belong to rare public or common public compartments (see above), there are five TCRβ sequences which are considered private in the current analysis.

**Table 1:** TCRβ CDR3 nucleotide sequences shared with other published works.

Publication	CDR3 nucleotide sequence	Clonal compartment in current analysis
Mwangi et al. (2010)	TGTGCCAGCAGTTTGGACAGGGGGATCATTTTO	Private
	IGTGCCAGCAGTGGGACAGGGGGATACAGTAACATGATTTTC	Private
	TGTGCCAGCAACAGGGGGATCGATATCCAGTATTTT	Private
	TGTGCCAGCAGTTTACAGGGACACACACCACTGAACTTT	Private
	TGTGCCAGCGGGACAGGGGGATACACACCACTGAACTTT	Rare publics
	IGCGCTAAGCAAGCGGGACAGGGCGAAAGACTGATCTTT	Rare publics
	TGTGCCAGCAGTCAGACAGGGGGATACACACCACTGAACTTT	Rare publics
	TGTGCCAGCAGTTCGACAGGGGGATCGTACAGTAACATGATTTTC	Rare publics
	TGTGCCAGCAGTTGGACAGGGGGAAACACACCACTGAACTTT	Rare publics
	IGTGCCAGCAGTAACCGGGACAGGGGGATCGAAAGACTGATCTTT	Rare publics
	TGTGCCAGCAGTTTGGACAGGGGGAGTAACATGATTTTC	Common publics
	TGTGCCAGCAGTTGGGGGATCAGTAACATGATTTTO	Common publics
	TGCGCTAAGACAGGGGGATACAGTAACATGATTTTO	Common publics
	TGTGCCAGCAGGACAGGGGGTAACATGATTTTC	Common publics
Mwangi et al. (2011)	TGTGCCAGCAGTTTGGACAGGGGGAGTAACATGATTTTC	Common publics
Zhang et al. (2020)	TGCGCTAAGCAAGTCGGGACAGGGATTAATATCCAGTATTTT	Private
	IGCGCTAAGCAAGATCGTAATATCCAGTATTTT	Rare publics

Sequence identity based on clonal compartments within current analysis is specified based on the specific private or public compartments. One sequence was found to be shared between the datasets and is highlighted in red.

### TCRβ V family usage and contributions to public compartments

**Figure 7:**
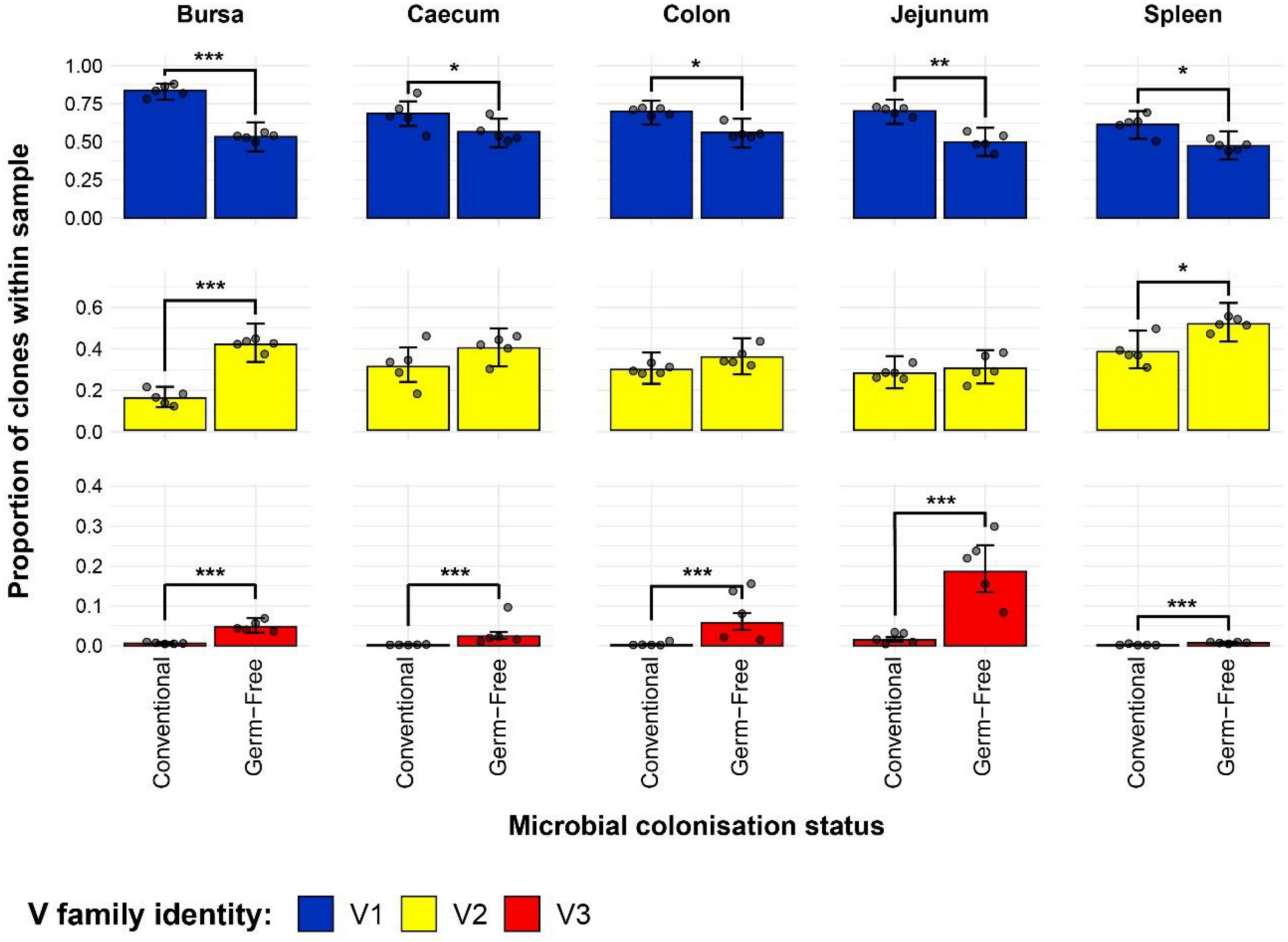
TCRβ V family usage in individual tissue samples across treatment groups. V family identities are shown in blue (Vβ1), yellow (Vβ2), and red (Vβ3). Grey dots represent individual bird observations for each V family. Error bars represent 95% bootstrap confidence intervals for the point estimates generated from 1000 simulations of the model. Statistically significant differences between the model estimates are depicted above the plots based on their corresponding p-values: *p < 0.05; **p < 0.01, ***p < 0.001.

The results of the analysis on individual V family usage reveal several patterns across the different tissue samples from the microbial colonisation groups (**Figure 7**). First, there was a higher Vβ1 contribution in all tissues of conventional birds when compared to their germ-free counterparts. Second, when considering the recently described Vβ3 family (39), all germ-free samples exhibited significantly higher contributions of Vβ3 clones to the TCRβ compartment, and these T cells are almost absent altogether from the tissues of the conventional birds, aside from the jejunum. Moreover, in the jejunal samples of germ-free birds, the Vβ3 clones were also found at the highest levels across all samples. Finally, only the bursal and splenic tissues show significant differences in the contribution of Vβ2 family to the overall TCRβ repertoire, with conventional birds exhibiting lower proportions than the germ-free counterparts.

**Figure 8:**
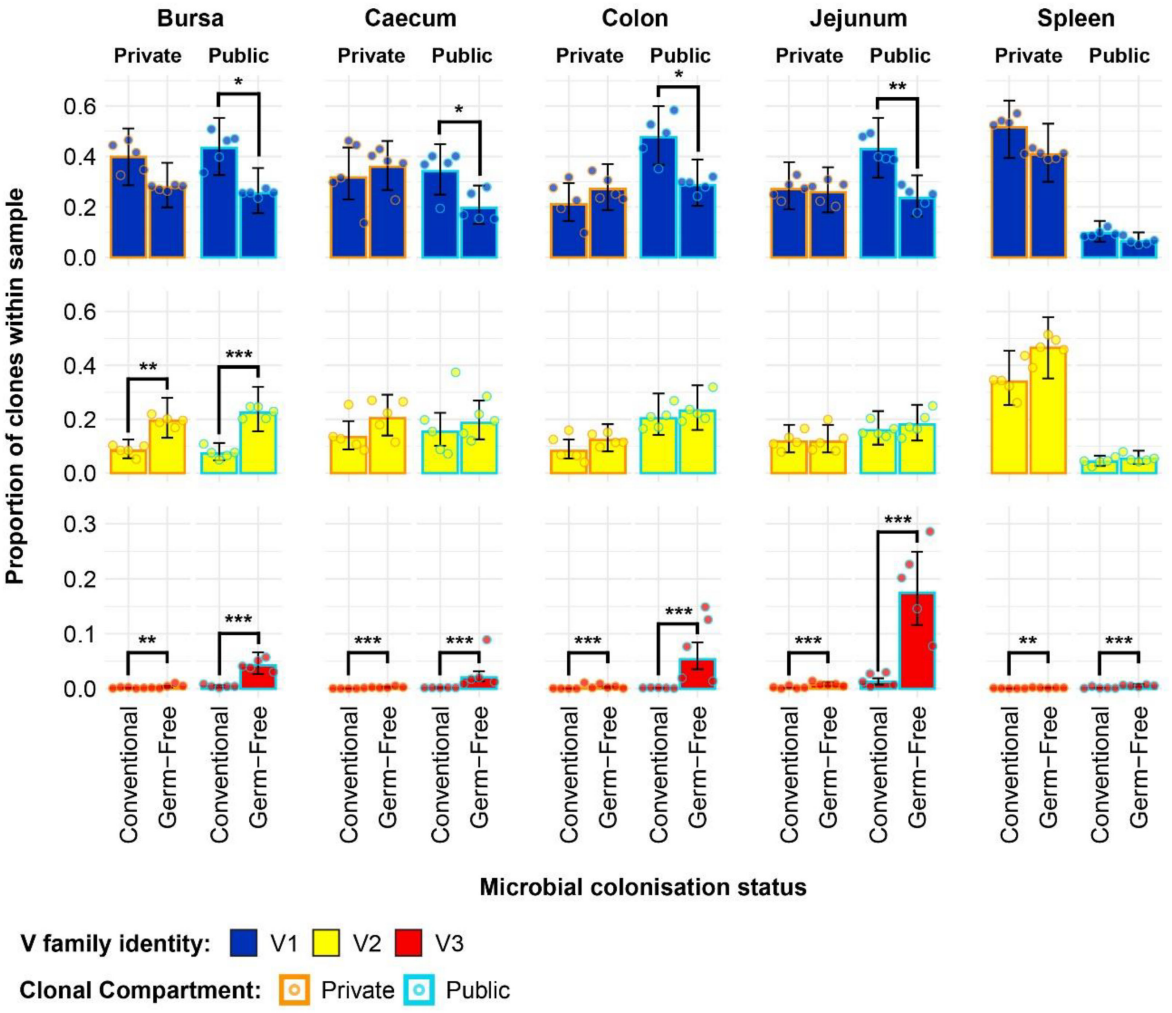
TCRβ clone V family publicness in individual tissue samples across treatment groups. V family identities are shown in blue (Vβ1), yellow (Vβ2), and red (Vβ3). Private (individual-restricted) clones have an orange outline. Public clones which are shared between more than two individuals have a light blue outline. Dots represent individual bird observations of V family contributions to the public and private clonal compartments. Error bars represent 95% bootstrap confidence intervals for the point estimates generated from 1000 simulations of the model. Statistically significant differences between the model estimates are depicted above the plots based on their corresponding p-values: *p < 0.05; **p < 0.01, ***p < 0.001.

By incorporating the distribution of V family clones in the private and public compartments, a more detailed pattern of the tissue-specific differences between the two gut microbiota colonisation groups is revealed (**Figure 8**). The overall higher Vβ1 presence in the conventional chickens was predominantly attributed to Vβ1 public clones. As such, this clonal compartment has a higher Vβ1 presence in all tissues aside from the spleen where the difference between the model estimates was not deemed statistically significant. Although the overall Vβ1 proportion of the repertoire was higher in the spleen, there were no significant differences in terms of either the public or private clonal compartments between the treatment groups. At the same time, concerning the private compartments, there were no significant differences in Vβ1 family distribution between the two groups.

With the Vβ2 family, the only significant differences that related to microbial status were in the bursa, where both the public and private compartments of germ-free birds showed higher proportions of Vβ2 clones. This is consistent with the previous estimates of the simpler model and reveals that the higher levels of Vβ2 in the germ-free bursa are similarly distributed between the public and private clonal compartments. For the spleen, the higher levels of Vβ2 clones in the germ-free which were observed with the initial model seem to be attributable to the private clonal compartment, although the estimates for the private and public clonal compartments of the two groups are not significantly different from one another.

**Figure 9:**
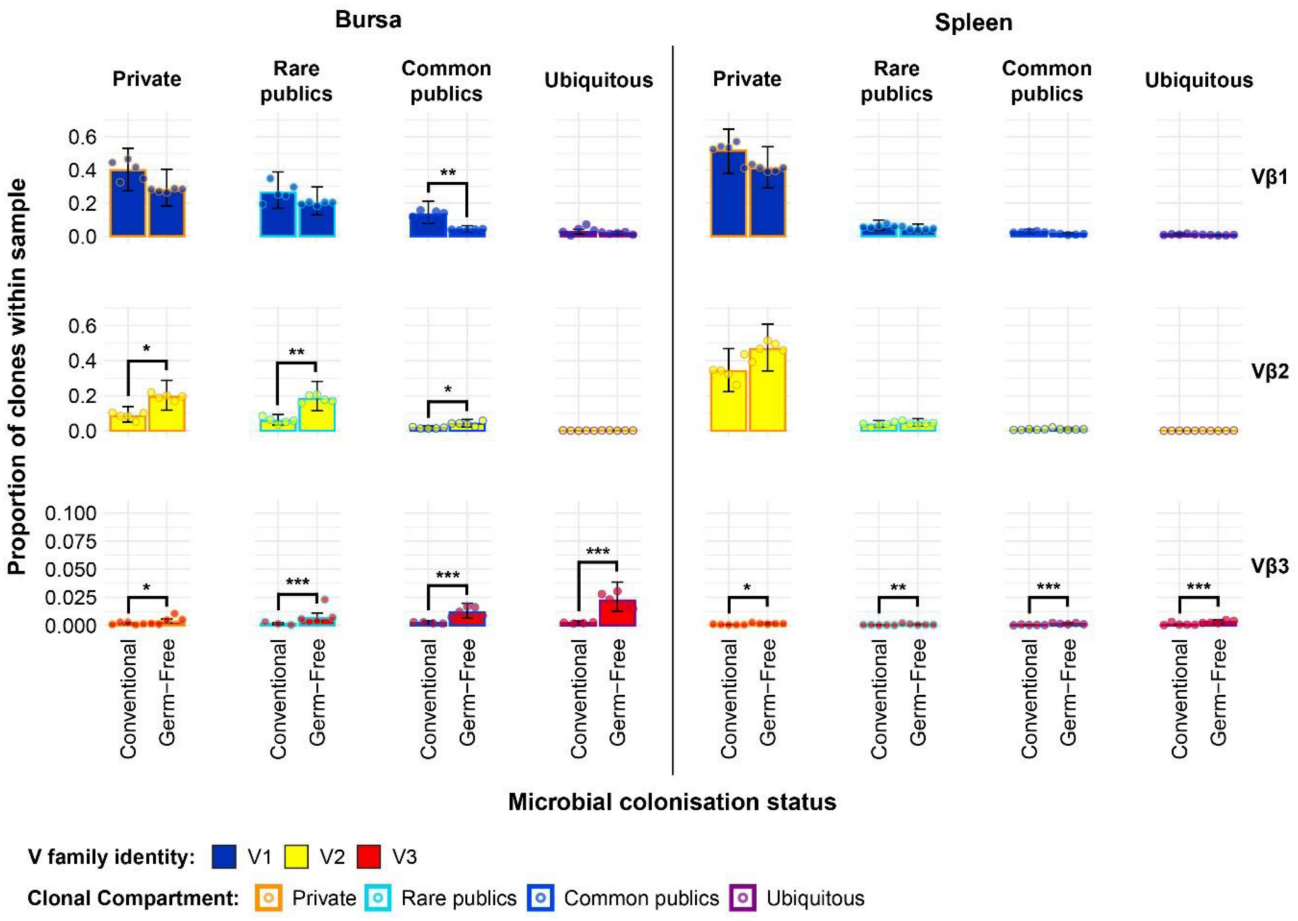
TCRβ clone V family private and public compartments in the bursa and spleen based on different levels of clonal sharing between birds. Private (individual-restricted) clones are outlined in orange. Rare publics (shared between ≥2 and up to 5 birds) and are outlined in light blue. Common publics (shared between ≥5 and up to 9 birds) are outlined in dark blue. Ubiquitous publics (found in all birds which were incorporated in the analysis) are outlined in purple. V family identities are shown in blue (Vβ1), yellow (Vβ2), and red (Vβ3). Dots represent individual bird observations of V family contributions to the public and private clonal compartments. Error bars show the 95% bootstrap confidence intervals for the point estimates generated from 1000 simulations of the model. Statistically significant differences between the model estimates are depicted above the plots based on their corresponding p-values: *p < 0.05; **p < 0.01, ***p < 0.001.

The incorporation of the public and private clonal compartments showed several interesting patterns for the Vβ3 family. First, Vβ3 clones are at very low levels in all birds, except for the germ-free jejunum where they are most abundant. Second, the germ-free birds have a significantly higher presence of Vβ3 clones in both the private and public compartments for all tissues. Third, the much higher relative levels of Vβ3 clones revealed by the initial model in the germ-free birds are predominantly public. The distribution of V family clones in the private and public compartments warranted further investigation by considering the degrees of clonal sharing. As such, the model was expanded to incorporate the different categories of public clones as described above. This revealed several differences at the levels of clonal compartment, tissue, and microbial status. In the spleen, there were no microbial status-driven differences between any of the clonal compartments within Vβ families, with the notable

exception of the Vβ3 family of the germ-free, which exhibited higher levels in the private, rare public, common public, and ubiquitous clones (**Figure 9**). In the bursa, the previously identified higher level of Vβ1 public clones in the conventional chickens was mainly attributable to the common publics, as no significant differences were present between rare public or the ubiquitous clones of the two groups. By contrast, the greater total Vβ2 public compartment of the germ-free group is attributable to both rare and common publics, both of these compartments exhibiting higher levels than in the conventional birds. At the same time, as observed in the spleen, the Vβ3 bursal clones were relatively more abundant in the germ-free group in all public compartments, albeit at very low levels.

**Figure 10:**
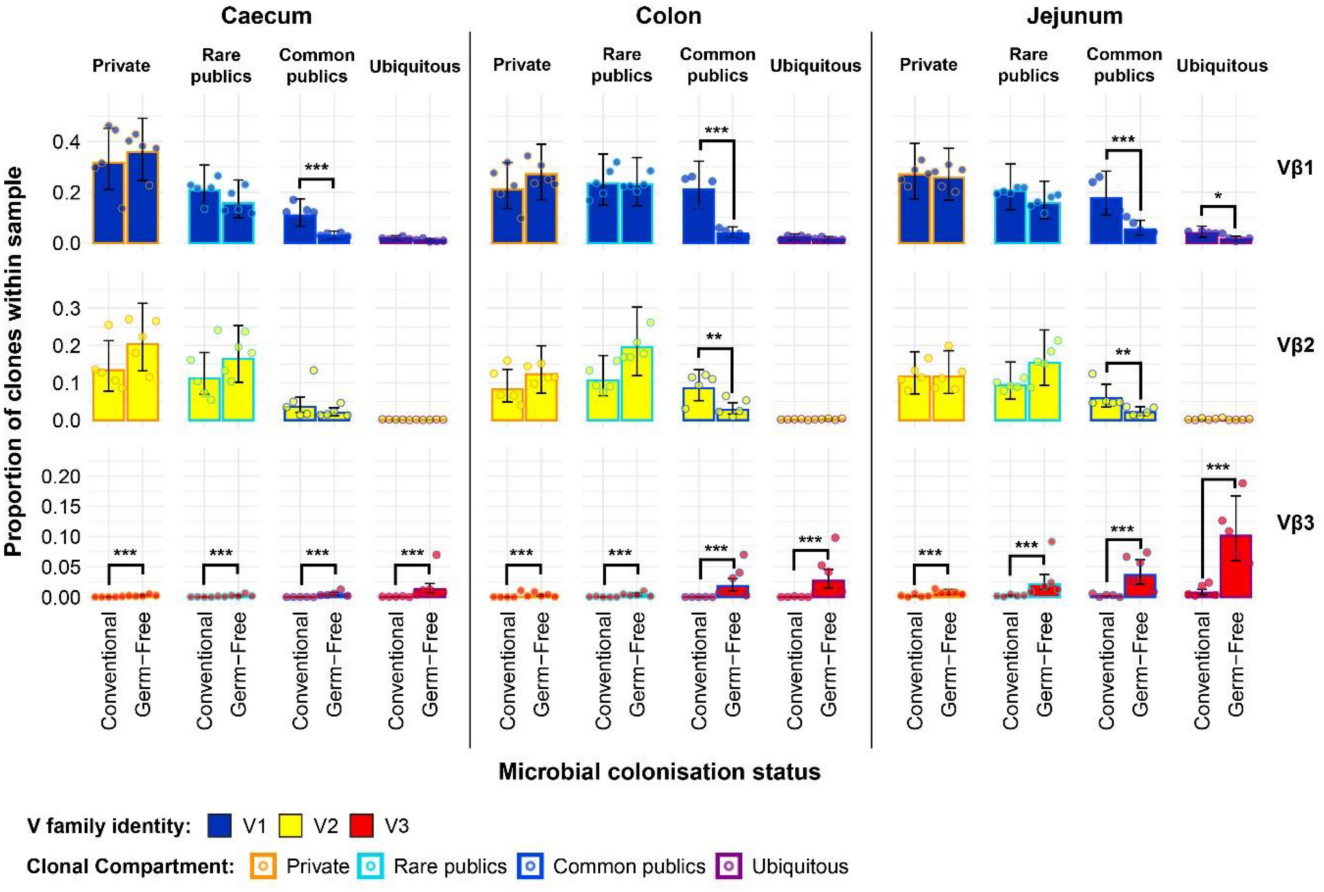
TCRβ clone V family private and public compartments in the intestinal tissues based on different levels of clonal sharing between birds. Private (individual-restricted) clones are outlined in orange. Rare publics (shared between >2 individuals up to 5) and are outlined in light blue. Common publics (shared between >5 and up to 9 birds) are outlined in dark blue. Ubiquitous publics (found in all birds which were incorporated in the analysis) are outlined in purple. V family identities are shown in blue (Vβ1), yellow (Vβ2), and red (Vβ3). Dots represent individual bird observations of V family contributions to the public and private clonal compartments. Error bars show the 95% bootstrap confidence intervals for the point estimates generated from 1000 simulations of the model. Statistically significant differences between the model estimates are depicted above the plots based ontheir corresponding p-values: *p < 0.05; **p < 0.01, ***p < 0.001.

The expanded model also reveals important patterns in the intestinal samples (**Figure 10**). The higher presence of Vβ1 public clones in the conventional birds suggested by the simpler model can be mainly attributed to the common public compartment. At the same time, there was a small but significantly higher ubiquitous Vβ1 clone presence in the jejunal samples of conventional SPF birds. When considering at the Vβ2 family, although the simpler model did not reveal marked differences between the total public compartment of intestinal tissues, conventional birds exhibit significantly higher levels of Vβ2 common publics in the colon and jejunum. Notably, as seen in the bursa and spleen, the levels of Vβ2 ubiquitous clones were very low or absent altogether in all intestinal samples. The germ-free birds also exhibited higher levels of Vβ3 clones in all public clonal compartments, with the ubiquitous clones reaching the highest levels in all intestinal tissue samples.

Together, the results of the Vβ family models, including the incorporation of clonal compartments based on the degree of sharing between birds reveal patterns which were obscured in the global repertoire analyses. Importantly, the Vβ3 family is predominantly public and much more abundant in the small and large intestines of germ-free chickens as opposed to their conventional microbiota counterparts.

### TCRβ J gene usage and contributions to public compartments

When considering the distributions of Jβ clones in the total repertoire of the samples, no consistent patterns were observed in terms of the differences related to microbial status (**Supplementary Figure 6**). Significant differences were only observed in the bursa and jejunum, where the conventional birds differ from the germ-free. In the former tissue, the conventional birds contained a higher proportion of the Jβ1 and lower levels of Jβ3, whilst in the jejunum the conventional birds contained higher proportions of Jβ4 and less Jβ2 than in the germ-free. Similar observations can be made regarding the distribution of Jβ rearranged clones in the public and private compartments (**Supplementary Figure 7**). Few significant differences were present between the two microbial colonisation groups, without any consistent pattern. The colon of conventional birds exhibits higher levels of Jβ1 public clones and lower levels of Jβ2 private clones than the germ-free. In the jejunum, the conventional chickens have higher Jβ4 and fewer Jβ2 public clones than the germ-free.

### Microbial status-restricted repertoires and clonal expansions

As significant effects of both tissue type and microbial treatment were observed in the V family contributions to the private and public clonal compartments, specific clonal expansions in the two groups were examined further. Clones which were found at or above 0.5% of the total repertoire in at least one sample were considered to be expanded. Focus was put on clones which were expanded in at least two birds (i.e. public clones) of a microbial treatment group and absent or below the expansion threshold in all individuals of the other.

**Figure 11:**
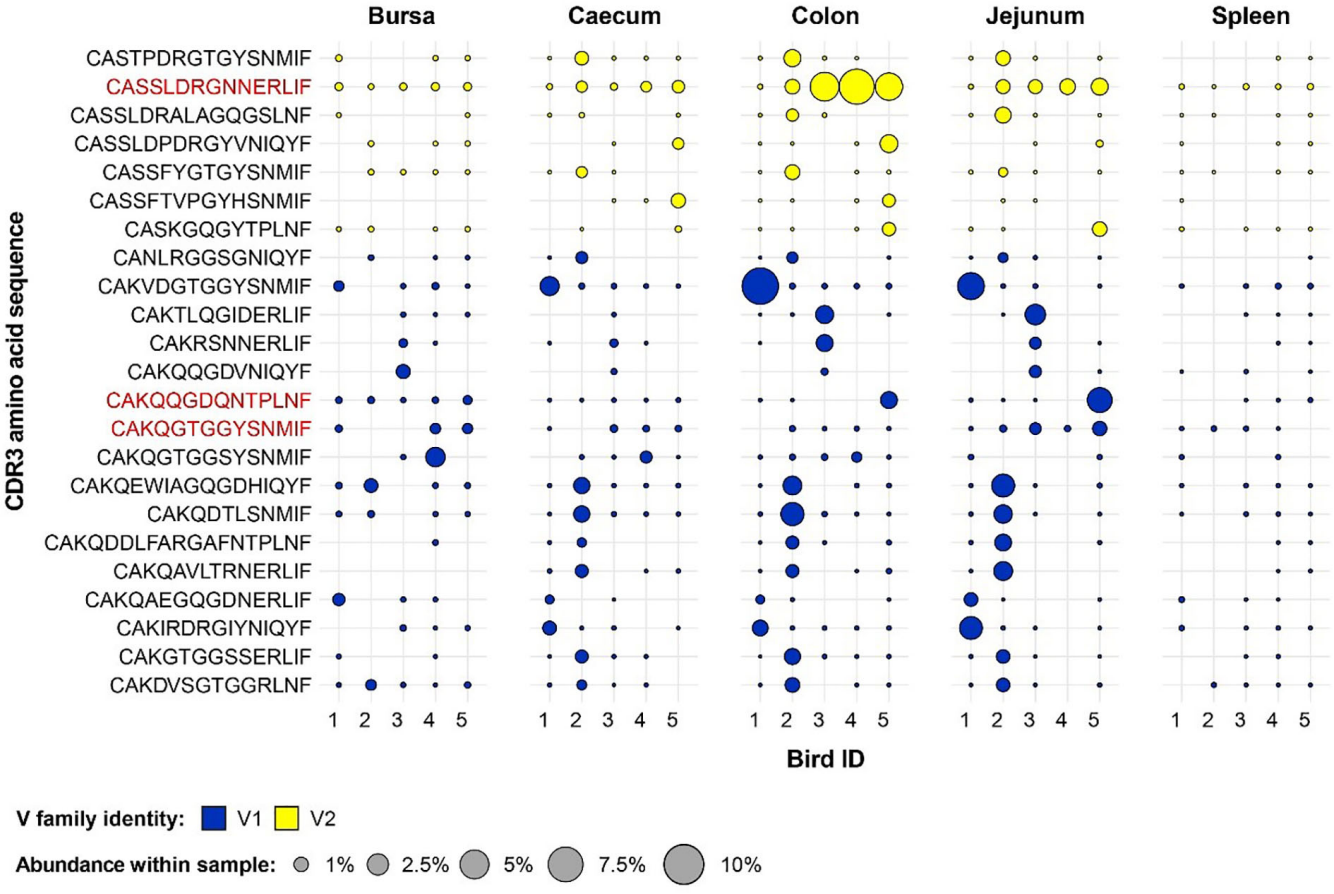
Clonal expansions in the restricted repertoire of conventional birds. Circles indicate the presence of clones with a specific CDR3 amino acid sequence and are proportional to the abundance within each bird’s tissue clonal compartment. The plot shows only the clones which are preferentially expanded in the conventional birds (at or above 0.5% and at less than 0.5% in the germ-free). Colours indicate V family identity: blue – V1 and yellow – V2. CDR3 amino acid sequences which are expanded in multiple birds are shown in red.

Several group-restricted expanded public clones were found in the conventional microbiota chicken samples with 16 belonging to the Vβ1 family and 7 to the Vβ2 (**Figure 11**). Interestingly, although the identified clones show a pronounced expansion in at least one sample, only 3 TCRβ clones were expanded across multiple birds, and none showed pronounced expansion in the spleen, where (if present) they were at low levels. Of these, two belong to the Vβ1 family and exhibit expansions in the bursa of several birds, one of which also showing patterns of expansions in the jejunum. The remaining Vβ2 clone which was expanded in multiple conventional microbiota birds shows marked levels (>5%) in 3 colon samples and is also present above 1% in 4 jejunal samples.

**Figure 12:**
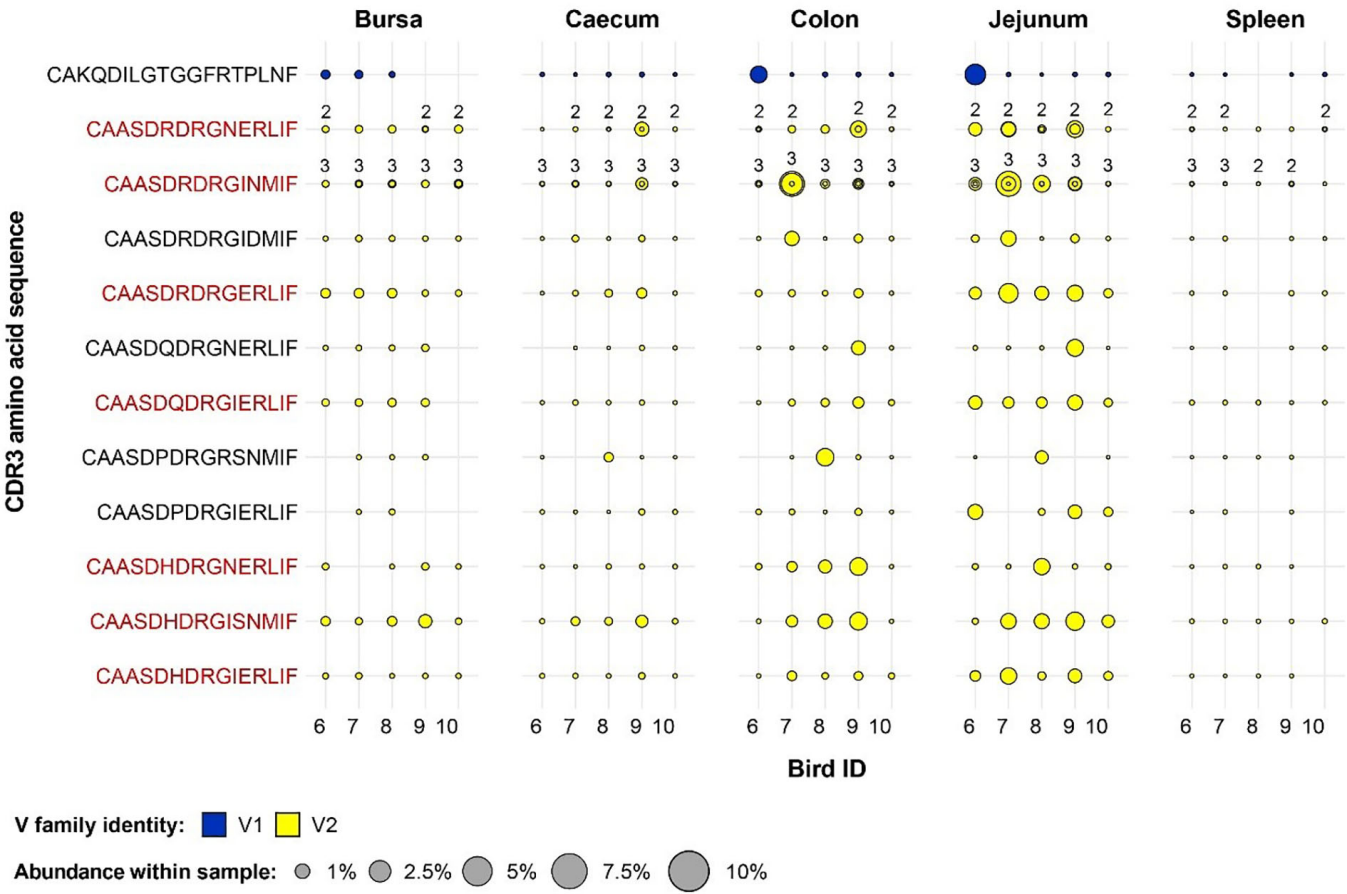
Clonal expansions in the restricted repertoire of germ-free birds. Circles indicate the presence of clones with a specific CDR3 amino acid sequence and are proportional to the abundance within each bird’s tissue clonal compartment. Overlapping circles display different clones based on nucleotide sequence which share the same CDR3 amino acid sequence. The number of convergent CDR3 nucleotide sequences recovered from the samples is displayed on top of the circles, if two or more are present. The plot shows only the clones which are preferentially expanded in the germ-free birds (at or above 0.5% and at less than 0.5% in the conventional). Colours indicate V family identity: blue – V1 and yellow – V2. CDR3 amino acid sequences which are expanded in multiple birds are shown in red.

A different pattern was observed when examining the germ-free restricted clones (**Figure 12**). Here, all but one of the 12 clones have a Vβ2 gene, whereas the remaining belongs to the Vβ1 family. Furthermore, out of the identified germ-free restricted and expanded TCRβ sequences, more than half show consistent patterns of expansion across multiple tissues from different birds. Importantly, the CDR3 amino acid sequences CAASDRDRGINMIF and CAASDRDRGNERLIF show convergence of 3 and 2 distinct clonotypes by nucleotide sequence, respectively. All these clones belong to the Vβ2 family, and exhibit marked levels of expansion, especially in the jejunum.

**Figure 13:**
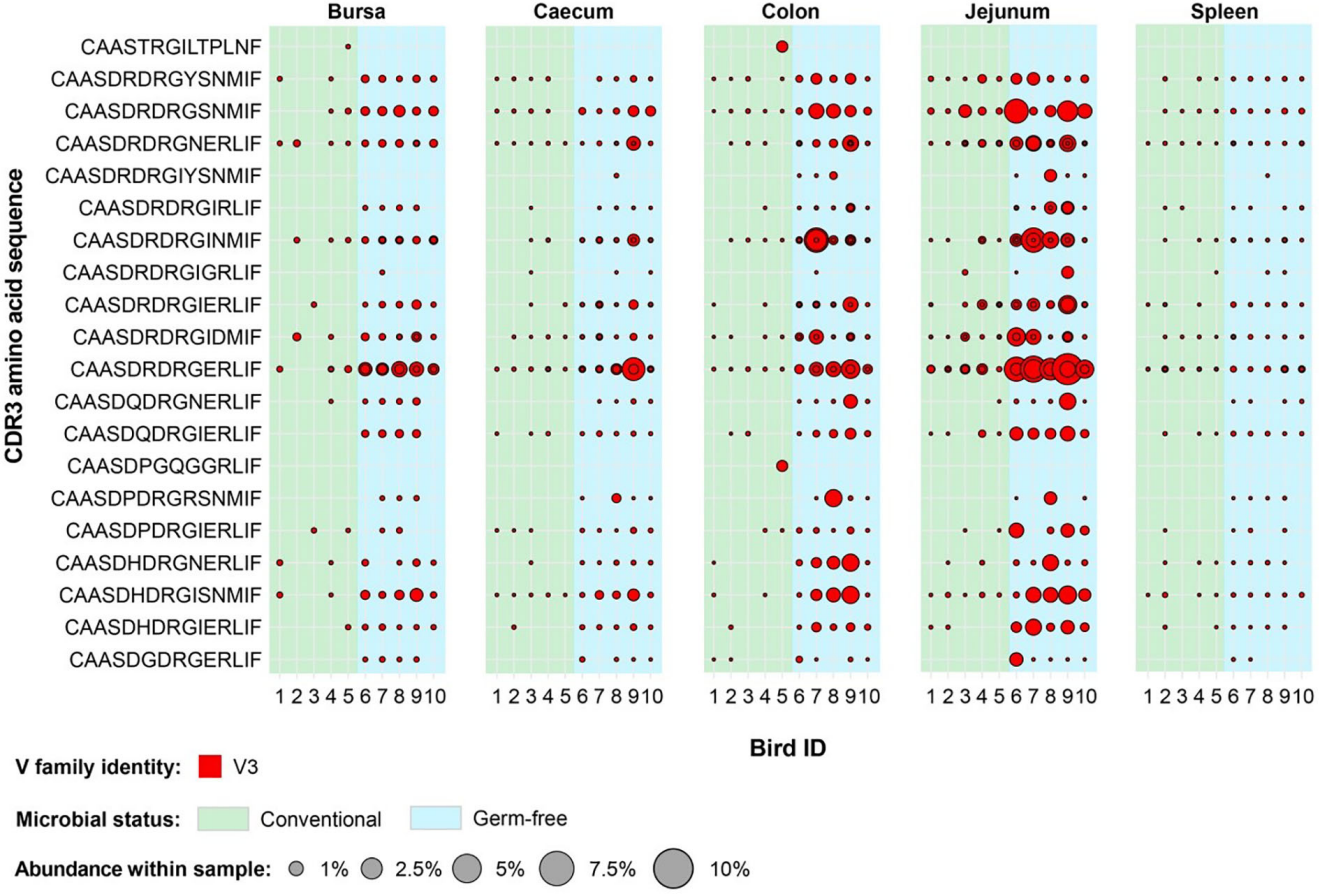
Clonally expanded Vβ3 CDR3 in tissues of germ-free and conventional birds. Red circles indicate the presence of clones with a specific CDR3 amino acid sequence and are proportional to the abundance within each bird’s (total) tissue clonal compartment. Overlapping circles display different clones based on nucleotide sequence which share the same CDR3 amino acid sequence. The plot depicts Vβ3 clones which are found at expanded (at or above 0.5%) in any of the birds included in this analysis (both germ-free and conventional). Background colours indicate microbial status group identity: green – conventional, light blue – germ-free.

Interestingly, for the TCRVβ3 repertoire, no clonal expansions were either restricted to germ-free or conventional groups with many clones present in a range of tissues in all or almost all birds (**Figure 13**). In a number of cases, the overall proportion of the expanded TCRVβ3 clones were greater in germ-free compared with conventional birds (e.g. with the CDR3: CASSDRDRGERLIF). The level of representation of this CDR3 (comprising two expanded clones at nucleotide sequence) in the jejunum was between 3 and 6% of the total TCRVβ repertoire in all germ-free birds. Although the CDR3 were variable in length we noted that in a central portion of the CDR3 a DRG motif was present in 18/20 expanded TCRVβ3 clones. The DRG motif is derived from one of the three D segment open reading frames. Interestingly the DRG motif was also overrepresented in TCRVβ2 germ-free specific expanded clones (**Figure 12**) but not in the expanded clones seen in TCRVβ1 or TCRVβ2 in conventional birds. We therefore considered the proportions of CDR3 amino acid clones that encode the DRG motif in germ-free or conventional birds for each TCRVβ family and each tissue (**Supplementary Figure 8**). The DRG motif is overrepresented in TCRVβ3 and is particularly prominent in the jejunum and other intestinal tissues. When we consider all unique clones of each Vβ family (i.e. not considering clonal expansions), for TCRVβ1 and TCRVβ2 CDR3 those containing the DRG motif was represented in ~10% of the CDR3 whereas for TCRVβ3 this sequence was identified in ~50% of the CDR3 (**Supplementary Figure 9**).

## Discussion

The microbiota is an important driver of the innate and adaptive immune systems, particularly in the gut and other mucosal tissues (7). Most studies have been focussed on mammalian systems and to consider the phylogenetic breadth of such interactions we chose to study the chicken. While relatively few studies have attempted to evaluate the effects of the gut microbiota on gut-associated lymphocytes in chickens, these do suggest important roles of the commensal microbiota in the development and normal functioning of the immune system (2, 4, 40, 41). The function of the adaptive immune system is dependent upon the structure of the repertoire of those receptors that recognise antigens. As with other species, TCRαβ+ T cells are a key part of the chicken immune system having both direct and indirect effects on immunity against pathogens (10). TCRβ-based repertoire analysis provides a key tool to determine the structure of the TCRαβ+ T cell populations including the basal available diversity as well as clonal bias/expansions according to circumstance. To consider the impact of microbial status on the repertoire of TCRαβ+ T cells a high-throughput sequencing (HTS) approach was adopted to interrogate multiple tissues of chickens reared under conventional or germ-free conditions. This HTS-based analysis provided fundamental insights into the core chicken TCRβ receptor repertoire at an unprecedented level and revealed that the microbiota shapes the diversity and composition of tissue-specific T cell compartments.

The observed diversity patterns are broadly consistent with previous results which have illustrated that the presence of microbiota drives clonal expansions that are otherwise not present under germ-free conditions (14). The current HTS analysis revealed a range of novel features of the chicken TCRβ population. For example, we were able to determine differences in TCRβ repertoire between tissues at the level of CDR3 sequence identity. The highest levels of clonal expansions were found in the gut segments, in particular those that relate to the large intestine (caecum and colon), and the highest diversity was detected in the spleen. As the large intestine contains the highest levels of microbial colonisation (and associated antigenic stimulus), this can be postulated as the factor that drives local clonal expansions in conventional birds. Indeed, the caecum is known to harbour the highest microbial densities of all intestinal segments (2) and the TCRβ repertoire in this tissue was the least diverse in birds reared with conventional microbiota. A very high proportion of rare clones was seen in the spleen of all birds irrespective of microbial status. As chickens lack conventional lymph nodes (10), our TCRβ repertoire data is consistent with the presence of a large population of naïve T cells in this tissue. The spleen may be an important organ for the generation of primary immune responses in the chicken. Indeed, even in mammals, primary immune responses can develop in the absence of lymph nodes (albeit with delayed dynamics) as evident in lymphotoxin alpha deficient mice (42).

Although the bursa has very few T cells [<4% of bursal lymphocytes (34, 35)], this organ is physically linked to the intestine (via the bursal duct) and the TCRβ diversity was influenced by microbial status. As the main site of B cell development, the function of the bursal T cells remains unknown. Previous studies indicated that the T cell infiltration and expansion occurs in response to challenge with Infectious Bursal Disease Virus (IBDV) (35, 43, 44). We hypothesise that the resident T cells might play other roles in relation to microbial antigens derived from the commensal microbiota of the gut. The presentation of microbially derived antigens by bursal antigen presenting cells, including B cells, might also promote the selected expansion of TCRαβ+ T cells which in turn might sustain and promote B cell expansions in relation to the microbiota within the gut. This topic deserves further investigation, and future studies might provide insight into the functional importance of bursal T cells under both physiological and pathological conditions.

The high degree of clonal sharing between birds (publicness) was a striking feature of the TCRβ repertoires as were the tissue specific differences in the levels of publicness. More than half of the total clones in the intestinal tissues and the bursa were shared between different individuals. Given the high theoretical diversity which can be generated during TCR rearrangement and the fact that the realised repertoire in any individual is restricted by the total number of T cells, a high degree of clonal sharing between different individuals is unlikely by chance (45). However, previous studies in rodents and humans have also revealed that the degree of TCRβ sharing between individuals can amount to a significant percentage of the total repertoire. In one study, close to 4% of human TCRβ CDR3 nucleotide sequences are shared between two individuals, and up to 40% when considering the amino acid level (46). In chickens, the current analysis indicates that almost 50% of TCRβ CDR3 nucleotide sequences were public in the gut tissues and in excess of 10% were public in the spleen. The differences in levels of publicness between tissues (spleen versus gut) has not been reported previously and may reflect the intrinsic biology of gut resident T cells. Some of these clones were present across a wide range of tissues and expanded in gut tissues of conventional birds (e.g. the TCRβ CDR3 with the amino acid sequence CASSLDRGNNERLIF) which may represent a common public clone reactive against antigens delivered by the microbiota. It is noteworthy that there were also some, although fewer, expanded public clones in germ-free individuals and with Vβ3 there was a large proportion of public expanded CDR3s, most prominent in the germ-free intestinal tissues but also expanded in conventional birds. These public clones may represent TCRαβ+ cells reactive against food antigens or unconventional T cells that are clonally pre-expanded [as seen with CD1-restricted iNKT cells in humans (47)]. The birds used for the current analysis were from the PA12 line, which represent a closed flock “outbred” line of White Leghorn chicken. Hence the levels of publicness seen in the current analysis are not likely a consequence of high levels of inbreeding. Indeed, some of the clones identified in the present study were also found in the few other studies that analysed TCRβ sequences from different lines of chicken (although the studies were at a much lower depth than the present study) (14, 36, 37, 39). This suggests that the observed patterns of clonal sharing might relate to the intrinsic biology of the chicken TCRβ repertoire and could therefore apply more widely. Hence, there is a strong suggestion that the TCRβ repertoires of chickens are biased towards specific rearrangements, potentially through the VDJ recombination process itself, thymic selection or post-thymic clonal expansion in the peripheral tissues.

The chicken TCRβ locus contains only 16 Vβ segments across 3 families (11 Vβ1, 4 Vβ2, and 1 Vβ3). By contrast, humans have at least 57 functional Vβ segments and mice have up to 30 Vβ segments which can contribute to generating the repertoire diversity (48). The chicken TCRβ locus also contains only 4 Jβ segments as opposed to mice (12) or humans (13), and only 1 Dβ gene (mice and humans have 2) (49). Together, these features of the chicken genome result in less intrinsic potential for generating TCRβ CDR3 diversity which may partially explain the higher degree of clonal sharing between individuals. The high levels of clonal sharing may mean that the available anticipatory repertoire of chickens is less diverse than seen in mammals. This has potential to impact on the ability of chickens to combat pathogens and respond to vaccines. Where multiple individuals utilise a TCR with the same sequence, a pathogen that is selected as an TCR escape variant in one individual may have continued advantage once transferred to other individuals. This has important implications for both pathogen resistance and vaccination in chickens. At the same time, this may indirectly affect the evolution of pathogens, including those that may be zoonotically transferred from chickens to humans.

Other intrinsic biological features may contribute to the high levels of public clones which were observed in this study. Chickens are one of the species with a high number of circulating γδ T cells, and the abundance of these cells increases with age (10, 11, 50). By contrast, in mice and humans, the proportion of circulating of γδ T cells is much lower and the relative importance [and full spectrum of roles) for γδ T cells in different species remains to be determined (for reviews, see (51) and (52)]. Nonetheless, it may be that the αβ and γδ T cells work together in many circumstances and the magnitude of particular γδ populations may influence the level of publicness of αβ T cells. Indeed, there are well documented public αβ T cells which perform non-classical functions, and the relative importance of these unconventional cells may differ according to the vertebrate species in question (38). It is worth noting that within the γδ T cell populations in humans, mice and chickens there are a mixture of subsets with public (or hyper public) TCR (11). It might therefore be worth considering the repertoire structure of both αβ and γδ T cells as an important defining feature of cells with unconventional functional capability.

There is, however, another explanation for the observed patterns of TCRβ public clones and this relates to the chicken MHC (12, 53). Briefly, the chicken MHC is more compact than in mammals and a less diverse set of MHC genes is expressed per haplotype (often one dominant MHC class I and one dominant MHC class II gene). This restricted or “Minimal Essential MHC” (54) has functional consequences presenting a less diverse set of self-peptides in the thymus which could then restrict the repertoire of developing TCR αβ T cells. This may lead to a higher likelihood of similar TCRVβ CDR3 being selected in different individuals. Our results indicating that the spleen contained a lower fraction of public TCRβ CDR3 may not support this hypothesis, however it is important to note that the depth of sequencing needed to determine such effects may need to be much larger than achieved here. There may be intrinsic differences in the publicness and repertoire of T cells restricted to MHC class I (CD8+) and MHC class II (CD4+), but these cell populations were not separated in our current analysis. It is also worth mentioning that different MHC molecules in the chicken have been classified as fastidious or promiscuous based upon the breadth of peptides that they present [for a review, see (53)] which may influence the breadth and level of publicness seen in the chicken TCRβ repertoire.

Although public TCRβ sequences were identified in all samples, the highest levels were in the intestinal and bursal samples which suggests that tissue-specific factors play an important part in shaping the resident T cell compartment. Importantly, high levels of clonal sharing were identified in both germ free and conventional birds, indicating that the gut microbiota is not the sole driver of the patterns of publicness in these tissues. When incorporating the different degrees of clonal sharing, however, the common publics are more pronounced in the conventionally-reared birds, whereas the ubiquitous clones are more prominent in the germ-free individuals. The higher presence of ubiquitous clones in the germ-free may represent expanded T cells in response to food-derived antigens or unconventional T cell populations that respond to intrinsic stimuli. By contrast, the higher level of common publics (with lower degree of sharing) in the conventional chickens may reflect the broad spectrum of antigen-specific expansions which can occur under the influence of the gastrointestinal microbiota.

Non-classical T cells with invariant or very limited repertoires are known to occur in mice or humans and their CDR3s were often shared between individuals (55, 56). Although these cells have yet to be described in chickens, they are known to be more abundant in specific tissues, especially in mucosal tissues such as the gut (e.g. iNKT cells or MAIT cells). There, these invariant T cells are often considered to exhibit innate-like functions and some have been demonstrated to interact with non-polymorphic presentation molecules such as CD1 and can respond to microbial derived antigens (47). Interestingly, CD1 genes can be identified in the chicken genome which supports the proposal that nonclassical T cell subsets are likely to be present in birds (57, 58). With this in mind, the high percentage of public clones in the chicken intestinal tissues could be explained by the increased presence of unconventional invariant T cells in these sites.

Although the genetic and physiological mechanisms that result in the high degree of clonal sharing between birds are outside the scope of the current study, the patterns of clonal sharing deserve further investigation. Furthermore, some of the private sequences in this study were also detected in other datasets despite most being based upon much lower sequence depth than the current study. Hence the degree of publicity or clonal sharing is likely to be higher than reported here although for many of these we expect that these will represent lower level public TCR rather than those found at higher frequency in a greater proportion of birds. The issues and contributing factors surrounding TCR publicity are ill-defined in all vertebrates, and this is a topic that deserves more attention.

Microbial colonisation was found to markedly influence the distribution of tissue specific Vβ family clones and their patterns of clonal sharing but less so when grouped according to Jβ gene usage. The Vβ domain of the TCR contains the CDR1 and CDR2 loops required for interacting with the MHC during antigen presentation (45). By contrast, the retained parts of the J gene encode regions in the TCR involved in chain heterodimerisation and are not modified during rearrangement (45). As such, these functional attributes may be the main driver of the observed differences. The differential contributions to CDR3 diversity and the functional domains that the V and J segments encode may bias clonal selection and antigen-specific expansions towards particular Vβ rather than Jβ TCR rearrangements.

The Vβ1 clones were more prominent in the conventional microbiota birds, irrespective of tissue type, suggesting that T cells with Vβ1 rearrangements may preferentially respond to microbially-derived antigens. This is supported by the fact that the clonal expansions restricted to the conventional birds were predominantly identified within Vβ1. By contrast, the lower proportions of Vβ1 in the intestines of germ-free birds are balanced by a higher proportion of Vβ3 clones, almost exclusively public. This suggests that the Vβ3 rearranged TCR sequences may have a tissue-specific function, perhaps relating to mucosal homeostasis. Another interesting aspect is that the only differences in Vβ2 clones according to microbial status were in the bursa and spleen, with germ-free chickens exhibiting higher relative proportions than their conventional counterparts. Collectively, these results indicate that the different Vβ families in the chicken may be utilised in cells that may perform different (but overlapping) functions, at least according to the publicness and clonal structure of the TCRβ associated with each family. Whether these families contribute differentially to pools of conventional and unconventional TCRαβ+ T cells could be a profitable avenue for future exploration.

In conclusion, our deep sequencing approaches of the TCRβ repertoire in conventional and germ-free chickens identified some novel and important aspects of avian T cell biology. These included the high proportion of public clones and the tissue bias of such clones. The work also defined the impact of microbial status on the developing gut TCRβ repertoire and how microbes shape its diversity and features. Moreover, our results provide a basis for future studies on the chicken TCR repertoire and in developing comparative biological frameworks on the evolution and structure of the TCR repertoire.

## Data availability statement

Datasets are available at Dryad: Dascalu et al. (2022), CDR3 sequences of Germ-free and Conventional Chickens, Dryad, Dataset, https://doi.org/10.5061/dryad.tht76hf38.

## Ethics statement

Animal experiments performed with germ-free and conventional chickens were carried out in strict accordance with French and EU legislation. The experiments were approved by the “Ministère de l’éducation nationale, de l’enseignement superieur et de la recherche”, under the Protocol No. APAFIS#5833-20l60624l6362298 v3. The 3R principles of Reduction, Replacement and Refinement were implemented throughout. Samples from the experiment were made available within the collaborative EMIDA ERANET programme "Development of Immune Function and Gut Health" (DIFAGH).

## Author contributions

Conceptualisation and experimental design; SD, AS, BK, PV, SP, and AB. SD carried out the sample preparation and computational analysis and wrote the first draft of the manuscript. RD, SP, and SF assisted with sample preparation. MB, SP, and PF assisted with the computational work. AS supervised the project and MB and MI helped with the supervision. All authors contributed with interpretation of the results. All authors discussed and contributed to the final manuscript.
