# Supplementary material for: The influences of microbial colonisation and germ-free status on the chicken TCRβ repertoire
Source: Front Immunol. 2023 Jan 4;13:1052297. doi: 10.3389/fimmu.2022.1052297 (PMC9847582; doi:10.3389/fimmu.2022.1052297)
Supplement: Supplementary file 1 [file DataSheet_1.docx]

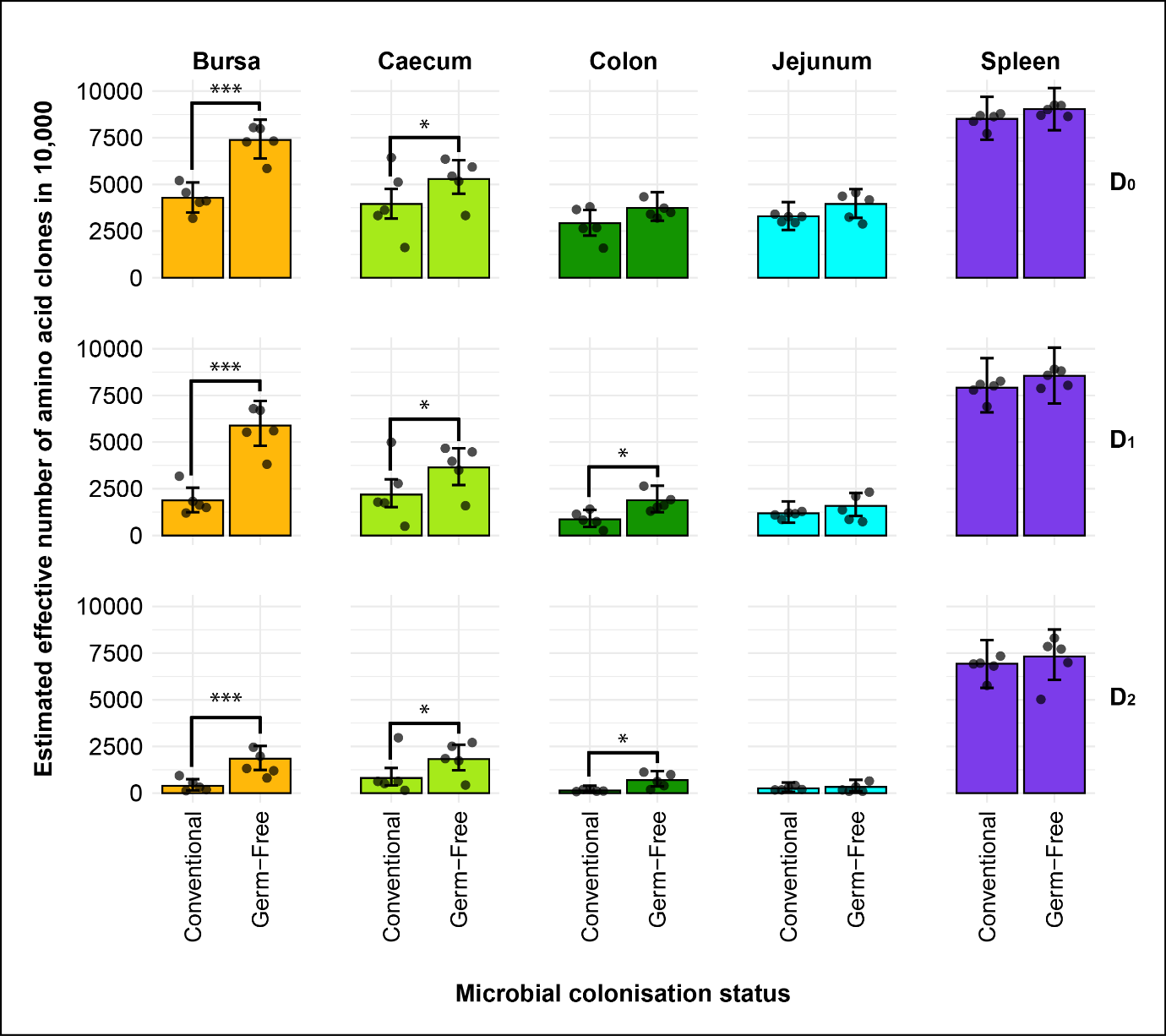


**Figure S1:** *Effective diversity within conventional and germ-free samples at the amino acid level*. Different rows show the effective number of clones corresponding to clonal richness (D_0_), the typical clones (D_1_), and dominant clones (D_2_). Tissues are colour coded for the bursa (orange), caecum (green), colon (dark green), jejunum (light blue), and spleen (purple). Dots represent individual bird observations of the effective number of species calculated in each tissue for the corresponding Hill number values. Error bars show the 95% bootstrap confidence intervals for the point estimates generated from 1000 simulations of the model. Statistically significant differences between the model estimates are depicted above the plots based on their corresponding p-values: * = p < 0.05; ** = p < 0.01, *** = p < 0.001.


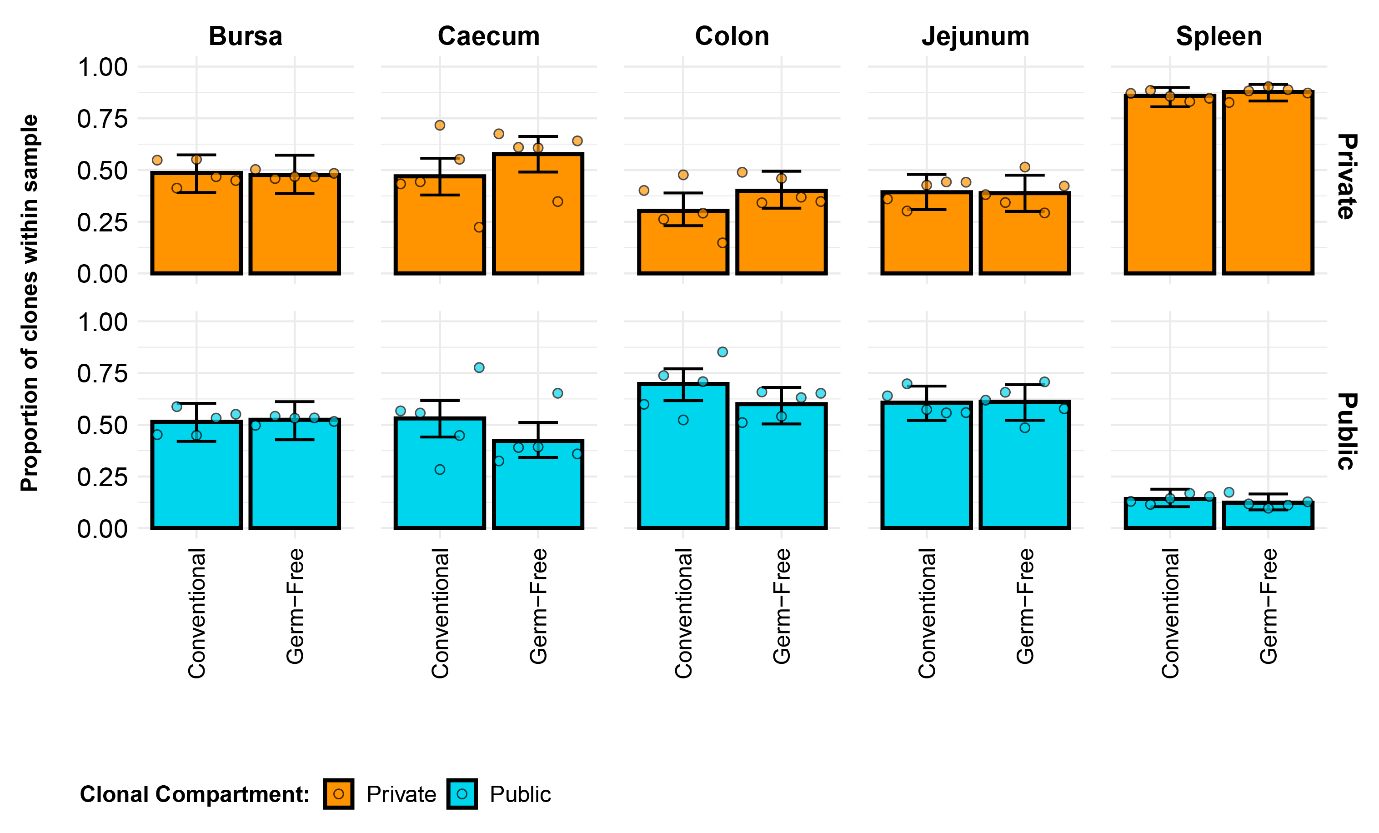


**Figure S2:** *Microbial status does not affect the proportional contribution of private and public CDR3 nucleotide sequences to the TCRβ repertoire.* Private (individual-restricted) clones are shown in orange. Public clones (shared between two or more individuals) and are shown in light blue. Dots represent individual bird observations of public and private clonal compartments. Error bars represent 95% bootstrap confidence intervals for the point estimates generated from 1000 simulations of the model. Statistically significant differences between the model estimates are depicted above the plots based on their corresponding p-values: * = p < 0.05; ** = p < 0.01, *** = p < 0.001.


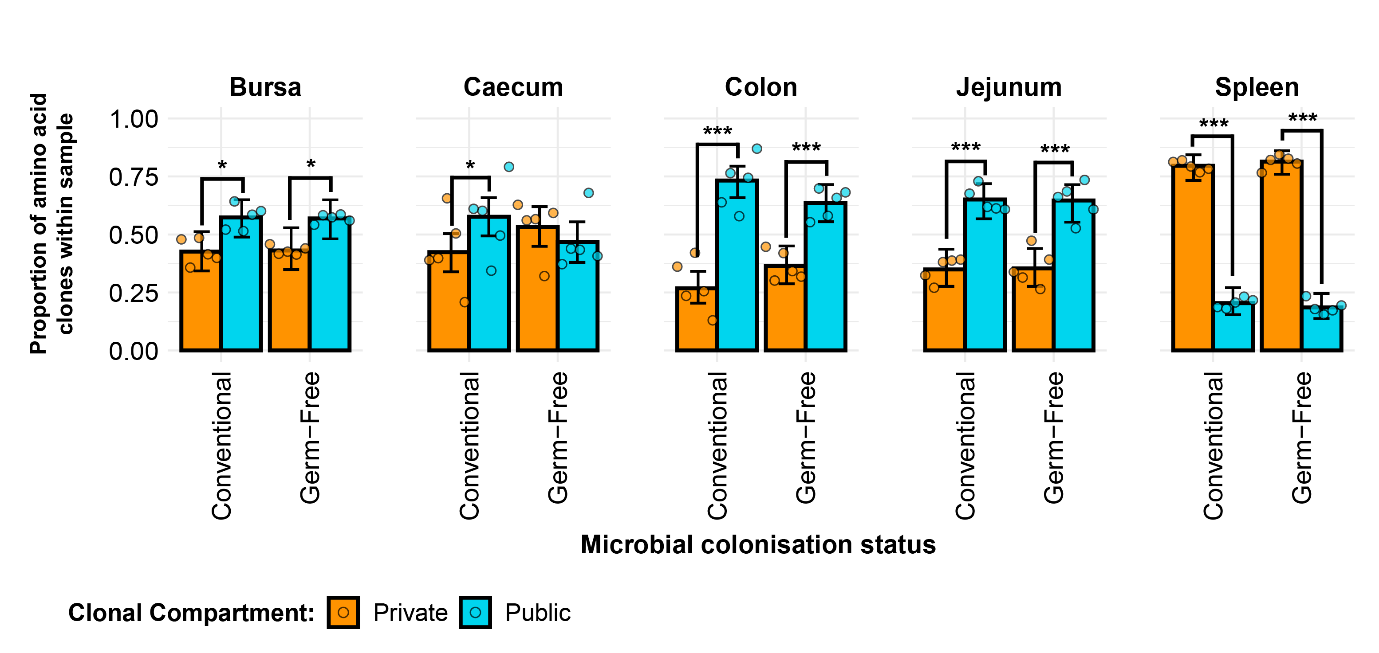
 **Figure S3:** *TCRβ clone CDR3 amino acid public and private compartments*. Private (individual-restricted) clones are shown in orange. Public clones (shared between two or more individuals) and are shown in light blue. Dots represent individual bird observations of public and private clonal compartments. Error bars represent 95% bootstrap confidence intervals for the point estimates generated from 1000 simulations of the model. Statistically significant differences between the model estimates are depicted above the plots based on their corresponding p-values: * = p < 0.05; ** = p < 0.01, *** = p < 0.001.


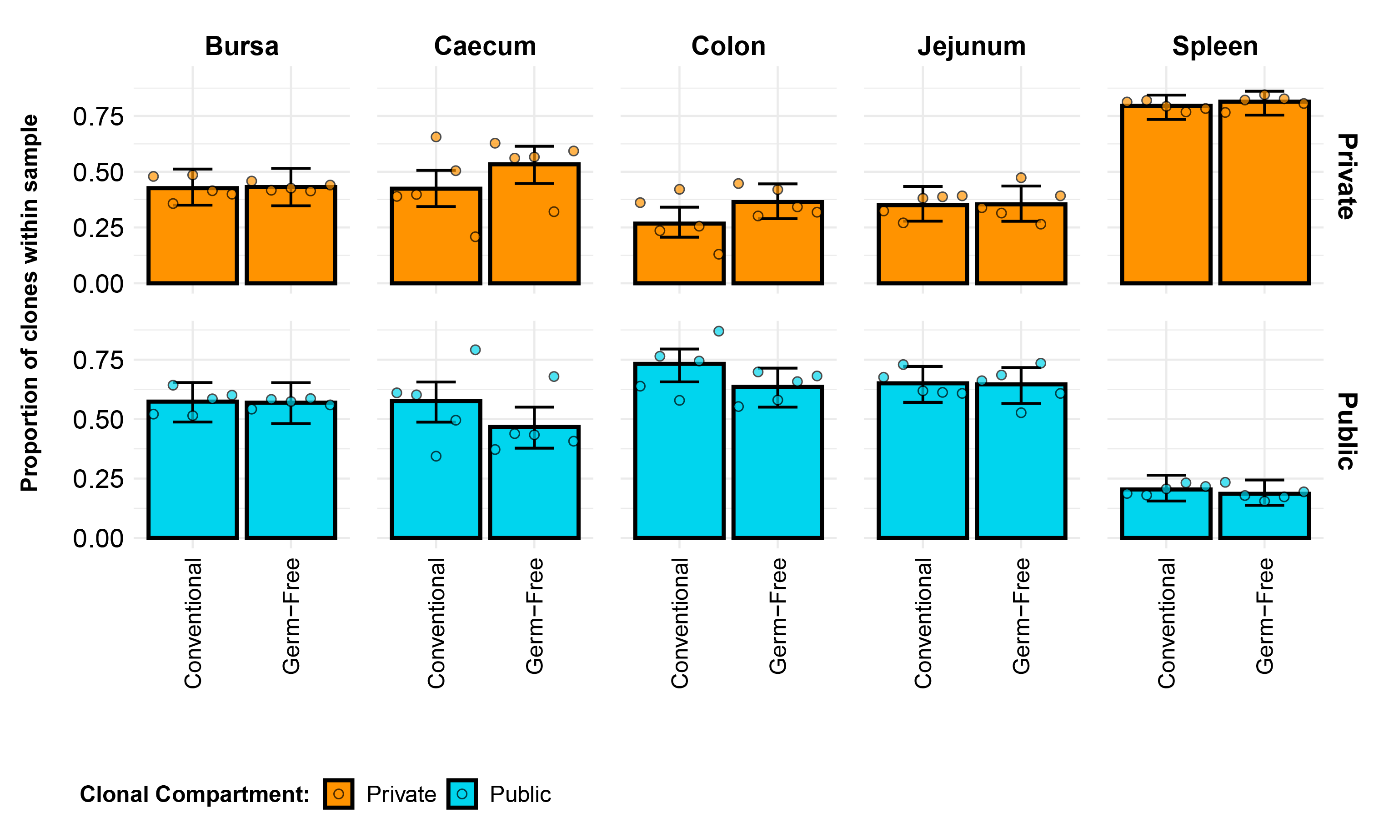


**Figure S4:** *Microbial status does not affect the proportional contribution of private and public CDR3 amino acid sequences to the TCRβ repertoire*. Private (individual-restricted) clones are shown in orange. Public clones (shared between more than two individuals) and are shown in light blue. Dots represent individual bird observations of public and private clonal compartments. Error bars represent 95% bootstrap confidence intervals for the point estimates generated from 1000 simulations of the model. Statistically significant differences between the model estimates are depicted above the plots based on their corresponding p-values: * = p < 0.05; ** = p < 0.01, *** = p < 0.001.


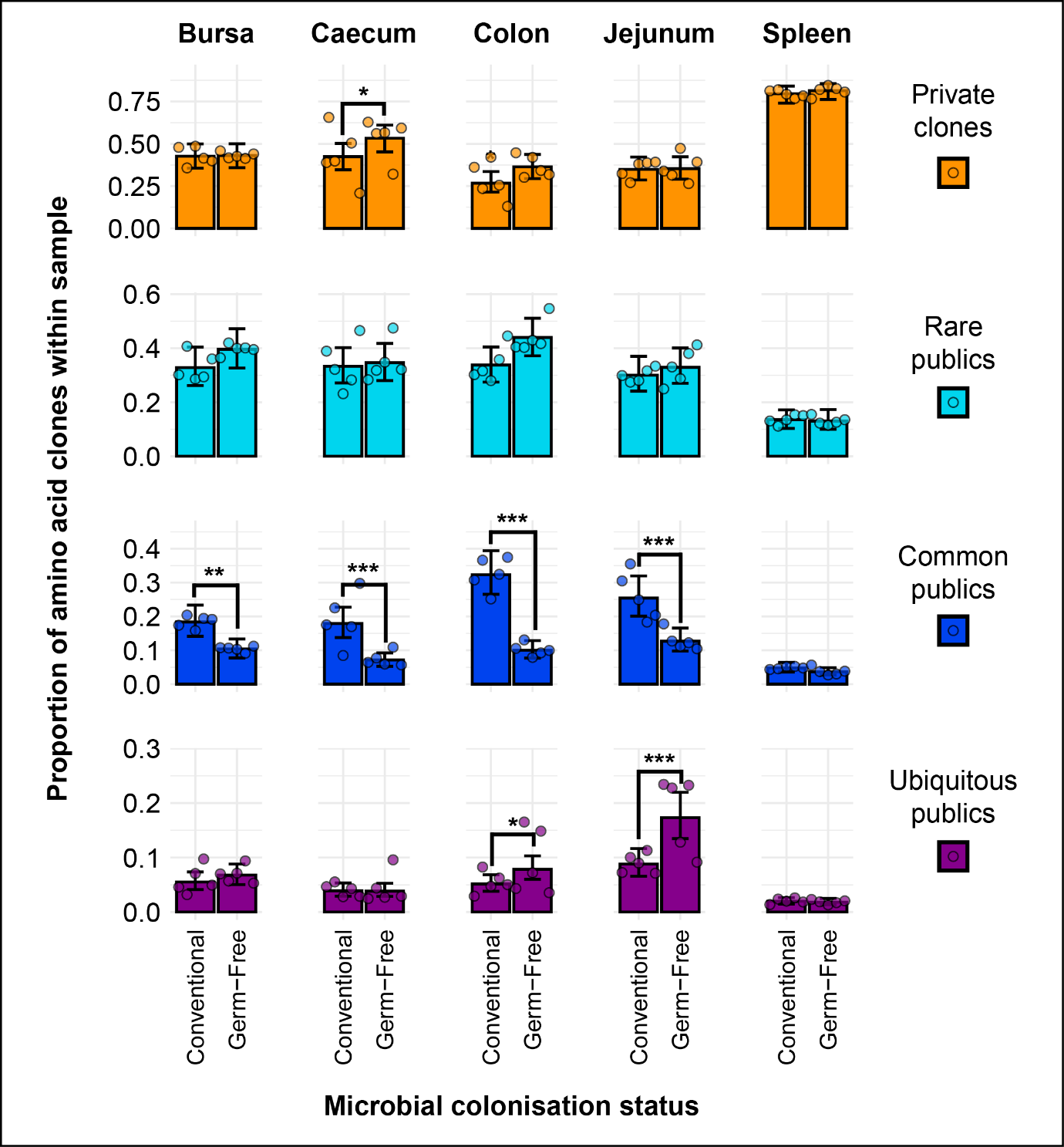


**Figure S5:** *TCRβ clone CDR3 amino acid private and public compartments based on different levels of clonal sharing between birds*. Private (individual-restricted) clones are shown in orange. Rare publics (shared between ≥2 individuals and up to 5) and are shown in light blue. Common publics (shared between ≥5 and up to 9 birds) are shown in dark blue. Ubiquitous publics (found in all birds which were incorporated in the analysis) are shown in purple. Dots represent individual bird observations of private and distinct public clonal compartments. Error bars represent 95% bootstrap confidence intervals for the point estimates generated from 1000 simulations of the model. Statistically significant differences between the model estimates are depicted above the plots based on their corresponding p-values: * = p < 0.05; ** = p < 0.01, *** = p < 0.001.


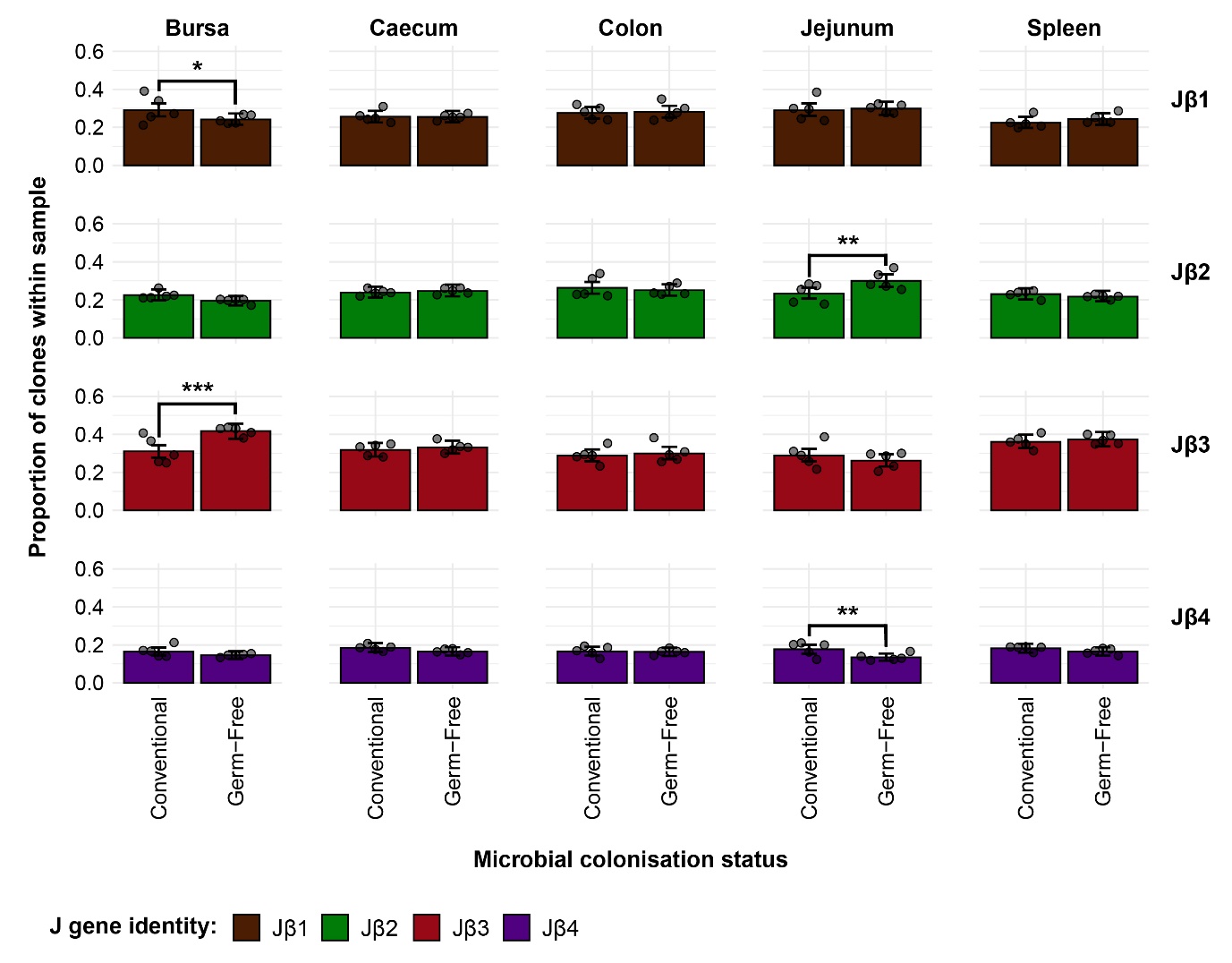
**Figure S6:** *TCRβ J gene usage in individual tissue samples across treatment groups*. V family identities are shown in brown (Jβ1), green (Jβ2), dark red (Jβ3), and purple (Jβ4). Grey dots represent individual bird observations for specific Jβ clones. Error bars represent 95% bootstrap confidence intervals for the point estimates generated from 1000 simulations of the model. Statistically significant differences between the model estimates are depicted above the plots based on their corresponding p-values: * = p < 0.05; ** = p < 0.01, *** = p < 0.001.


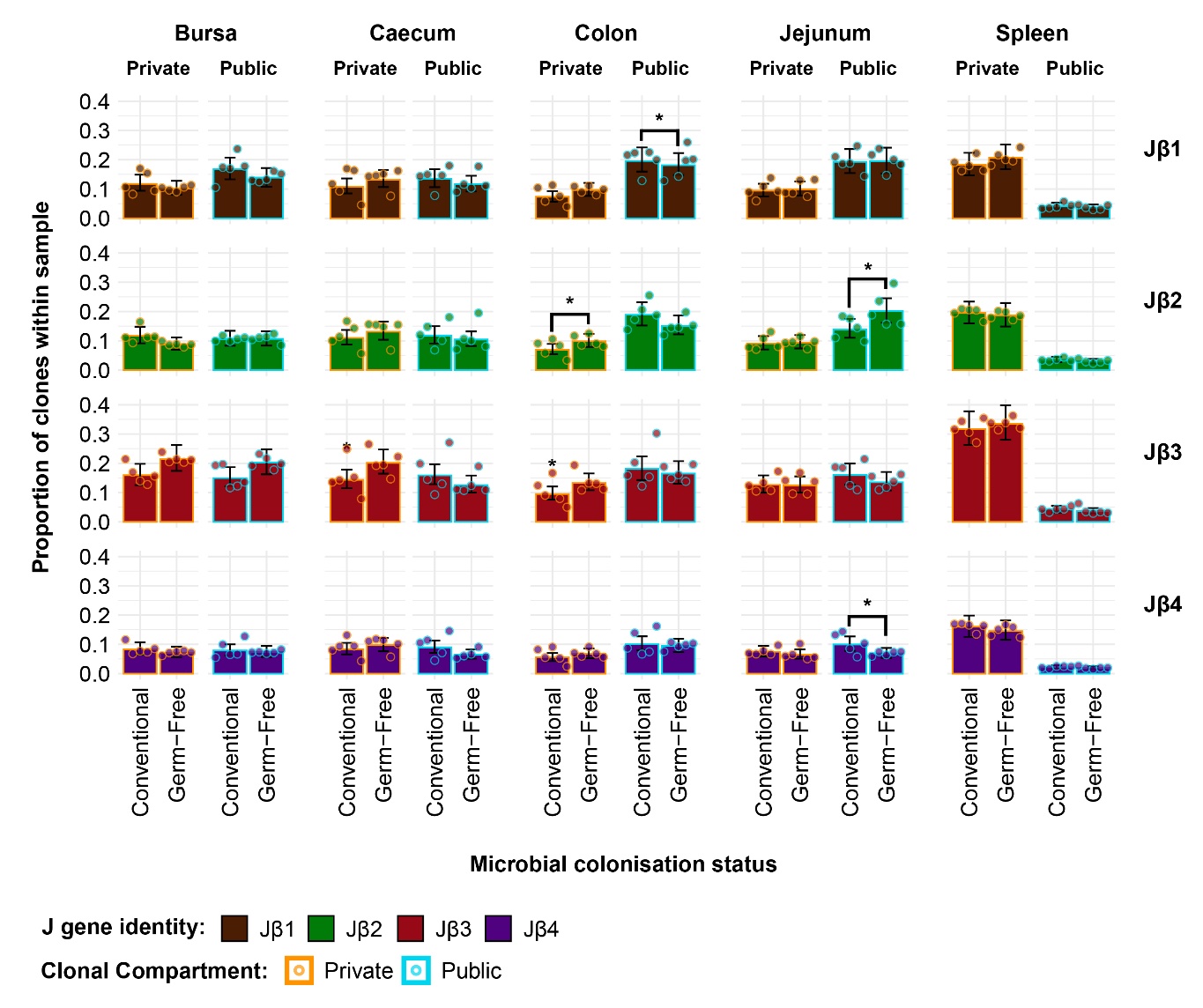
**Figure S7:** *TCRβ J gene publicness in individual tissue samples across treatment groups*. J family identities are shown in brown (Jβ1), green (Jβ2), dark red (Jβ3), and purple (Jβ4). Private (individual-restricted) clones have an orange outline. Public clones which are shared between more than two individuals have a light blue outline. Dots represent individual bird observations for specific Jβ clone contributions to the public and private clonal compartments. Error bars represent 95% bootstrap confidence intervals for the point estimates generated from 1000 simulations of the model. Statistically significant differences between the model estimates are depicted above the plots based on their corresponding p-values: * = p < 0.05; ** = p < 0.01, *** = p < 0.001.


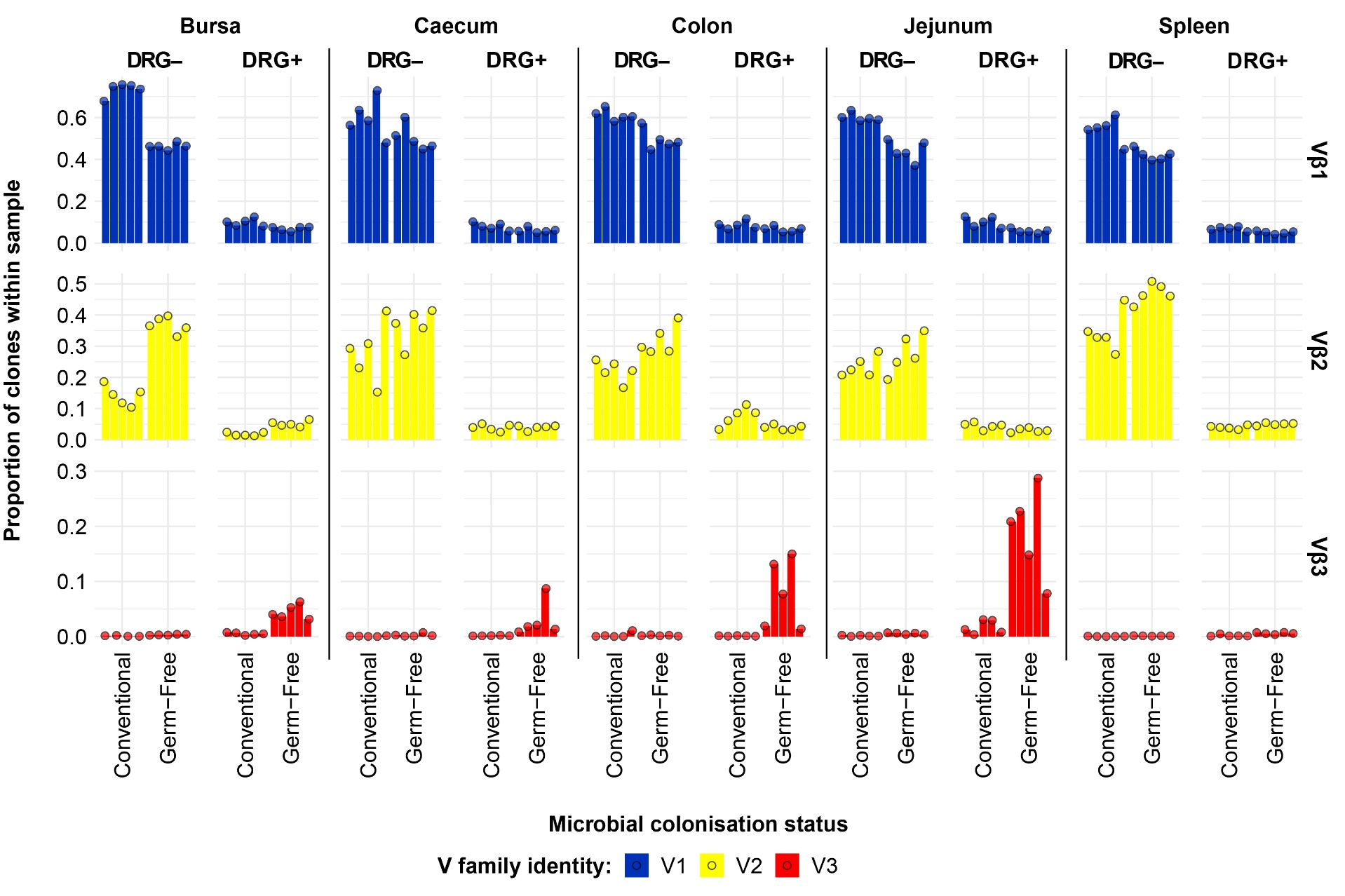


**Figure S8:** *Proportion of the repertoire occupied by clones with and without the DRG motif within their CDR3 by V family, tissue, and microbial status.* V family identities are shown in blue (Vβ1), yellow (Vβ2), and red (Vβ3). Dots and bars represent individual bird observations.

**Figure S9:** *Percentage of unique clones with the DRG motif within their CDR3 sequence.* The numbers are rounded to one decimal point for convenience. Abundance within birds and sample is not represented in the figure, as only the unique clones were considered.
